# Combining Spatial, Genetic, and Environmental Risk Data to Define and Prioritize In Situ Conservation Units

**DOI:** 10.1002/ece3.71251

**Published:** 2025-05-26

**Authors:** Eilish S. McMaster, Richard J. Dimon, Andrew G. Baker, Craig Harre, Justin Mallee, Aleks Maric, Peter Richards, Matthew Wiseman, Simon Y. W. Ho, Maurizio Rossetto

**Affiliations:** ^1^ School of Life and Environmental Sciences University of Sydney Camperdown New South Wales Australia; ^2^ Research Centre for Ecosystem Resilience Botanic Gardens of Sydney Sydney New South Wales Australia; ^3^ Queensland Alliance of Agriculture and Food Innovation University of Queensland St Lucia Queensland Australia; ^4^ Forest Research Centre School of Environment, Science and Engineering, Southern Cross University Lismore New South Wales Australia; ^5^ North Coast Aerial Mapping Coffs Harbour Arrawarra Headland NSW Australia; ^6^ New South Wales National Parks and Wildlife Service Alstonville NSW Australia; ^7^ New South Wales National Parks and Wildlife Service Kyogle NSW Australia

**Keywords:** conservation, in situ management, population genomics, threatened species

## Abstract

In situ management aims to preserve species and their genetic integrity within their natural habitat. To achieve this, conservation strategies must strike a balance between safeguarding genetic diversity, mitigating environmental risks, and addressing practical management constraints. Here, we present a clear and reproducible framework that addresses these goals.

We applied this framework to the Nightcap reserves in the Gondwanan Rainforests of Australia, a UNESCO World Heritage site impacted by the 2019/20 Black Summer fires. We analyzed the genetic diversity of 12 rainforest tree species, including three endangered species—*Eidothea hardeniana*, *Uromyrtus australis*, and *Elaeocarpus sedentarius*—and examined how fire risk, influenced by the presence of fire‐dependent species such as eucalypts, impacts genetic diversity.

To guide specific in situ management for the endangered species, we developed a flexible framework that uses clustering algorithms (DBSCAN and *k*‐means) to define spatial management units while considering resource limitations (e.g., maximum perimeter or area). Our framework also incorporates a composite genetic value metric (combining Essential Biodiversity Variables heterozygosity, allelic richness, and genetic differentiation) and evaluates future fire risk based on vegetation flammability. This approach allowed us to identify priority management areas while adhering to resource constraints.

We provide some reproducible examples of how the proposed framework can be applied, either partially or fully, to optimize in situ conservation efforts. Its flexibility allows for adjustments to fit different habitat types, species, and environmental threats, making it a valuable tool for enhancing conservation management across diverse conservation contexts.

## Graphical abstract

This study develops a flexible framework for in situ conservation using clustering algorithms (DBSCAN and *k*‐means) to define management units while considering resource constraints. It integrates genetic metrics (heterozygosity, allelic richness, differentiation) and fire risk assessments based on vegetation flammability to prioritize conservation areas. The approach is demonstrated with reproducible examples for optimizing conservation efforts.

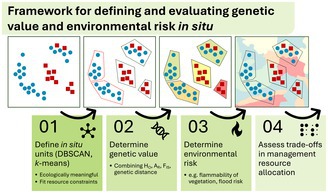

## 1 Introduction

A critical strategy for safeguarding biodiversity is in situ conservation, which aims to preserve self‐sustaining populations of endangered species within their natural habitats. This approach offers major advantages because it enables species to persist within the communities and ecosystems to which they have adapted over time, supports the complex interactions and ecological functions that sustain their survival, and allows ongoing adaptation (Braverman 2014; Heywood and Dulloo 2005; Whitlock et al. 2016). However, the success of in situ conservation is influenced by a range of factors, including resource availability, genetic diversity, and environmental risks (Heywood and Dulloo 2005; Maxted et al. 1997). Understanding and addressing these factors is essential for ensuring the long‐term effectiveness of conservation efforts.

The scope and scale of in situ management interventions determine what actions can be taken, and these actions are often constrained by both financial and practical limitations (Hoffmann 2022). Consequently, conservation managers are frequently forced to make qualitative assessments and manually define conservation sites, which can lead to inconsistent or suboptimal outcomes. While larger scale, systematic methods for defining conservation areas have been employed (Andrello et al. 2022; Burgess et al. 2019; Moilanen et al. forthcoming; Watts et al. 2009), such approaches are less common on smaller scales, where management decisions are typically driven by local knowledge and immediate resource availability. This limitation underscores the need for more robust and standardized methods to define conservation sites, allowing for more effective, evidence‐based, and equitable management.

Genetic diversity is another critical consideration for in situ conservation. Maintaining genetic diversity is integral to conservation because it enhances the resilience and adaptability of species (Sgrò et al. 2011), mitigates the risks of inbreeding depression (Frankham 2015; Reed and Frankham 2003), and supports broader ecological and evolutionary processes (Hoban et al. 2013; Hughes 2023; Vellend 2005). Global conservation policies, including the Post‐2020 Global Biodiversity Framework under the UN Convention on Biological Diversity (CBD), increasingly recognize the importance of genetic diversity in conservation (CBD/COP/DEC/15/4 2022). Experts have proposed various targets for maintaining genetic diversity, but the exact thresholds and relevant aspects of genetic diversity to prioritize are still not clearly defined (Frankham 2022; Hughes 2023; Laikre et al. 2020). To support the CBD's biodiversity goals, the Group on Earth Observations Biodiversity Observation Network developed the Essential Biodiversity Variables (EBVs)—a framework of four broad categories of genetic metrics (genetic diversity, differentiation, inbreeding, and effective population size) aimed at harmonizing and interpreting biodiversity data across studies (Hoban et al. 2022). However, challenges persist in evaluating genetic value across these diverse categories in a way that is both meaningful and applicable to in situ conservation goals (Díaz et al. 2020; Nielsen et al. 2017).

In addition to maintaining genetic diversity, environmental risks, particularly those that vary across space, play a substantial role in the success of in situ conservation. Factors such as wildfires (Collins et al. 2021), drought (Brunner and Chartier‐Rescan 2024; Yurkonis and Meiners 2006), pollution (Bartrons et al. 2016; Bobbink et al. 1998), and urban encroachment (Concepción et al. 2015) pose differential threats to species across their geographic ranges. These heterogeneous risks, often exacerbated by climate change and human activities, can undermine the effectiveness of conservation efforts if not properly assessed. Wildfires, in particular, have emerged as a critical concern, as anthropogenic climate change leads to more frequent and severe fires that threaten both ecosystems and biodiversity globally (Hao et al. 2018; Mukherjee and Mishra 2021). Failure to consider the varying risks faced by different conservation units could lead to ineffective or misdirected conservation actions.

In this study, we address these three key aspects of in situ conservation planning: the definition and evaluation of management units or sites, the challenges of managing genetic diversity across multiple, sometimes conflicting variables, and the integration of environmental risks into conservation strategies. Using a case study of rainforest tree species in the Nightcap Range in northern New South Wales, Australia, we explore how these factors intersect and shape in situ conservation planning, particularly on how fire can impact the genetic diversity of plant species.

This area, part of the UNESCO World Heritage‐listed Gondwanan Rainforests of Australia (UNESCO World Heritage Convention 1986), supports a unique assemblage of vegetation types, from fire‐dependent heathlands and sclerophyll forests to fire‐sensitive rainforest ecosystems (Crisp et al. 2009; Hill et al. 1999; Kooyman et al. 2014; Zachos and Habel 2011). The rainforest continues to support species that reflect major evolutionary stages from Gondwanan times (Kooyman et al. 2020; Zachos and Habel 2011), including several endangered endemic plant species. Among them are the federally listed Endangered *Uromyrtus australis* A.J. Scott, Critically Endangered *Eidothea hardeniana* P.H. Weston & Kooyman, and Endangered *Elaeocarpus sedentarius* Maynard & Crayn. The 2019/20 fires represented the largest recorded burn in the Nightcap Range, affecting rainforest areas that had remained unburned for over 1000 years (Benwell 2024; Kooyman 2023; Turner 1984) and raising concerns about future fire risks particularly for threatened endemic species.

Here, we develop an analytical framework that integrates genetic, ecological, and environmental data to evaluate the future risks posed by fire to biodiversity (represented by within‐species genetic diversity) in the Nightcap reserves and provide specific guidance for on‐ground fire mitigation. Using a high‐resolution vegetation map, satellite fire data from 2019/20, and population genetic data for 12 rainforest tree species, we aim to understand the distribution of genetic diversity across different vegetation types, how these types were affected by the 2019/20 fires, and how integrating practical constraints, genetic diversity, and future fire risks can inform the management of threatened species in situ and minimize the risk of genetic diversity loss.

The framework is adaptable to various contexts far beyond the example presented, enabling the integration of genetic and any environmental risk data to guide conservation planning for one or multiple species. By adjusting parameters, it can be customized to accommodate specific ecological characteristics, management goals (e.g., wildlife fence planning or land buyback programs), and environmental threats (e.g., drought, floods, urbanization, and agricultural encroachment). This flexibility makes the framework applicable for guiding broader conservation efforts and enhancing in situ management across diverse landscapes.

## 2 Methods

### 2.1 Study Site

**FIGURE 1:**
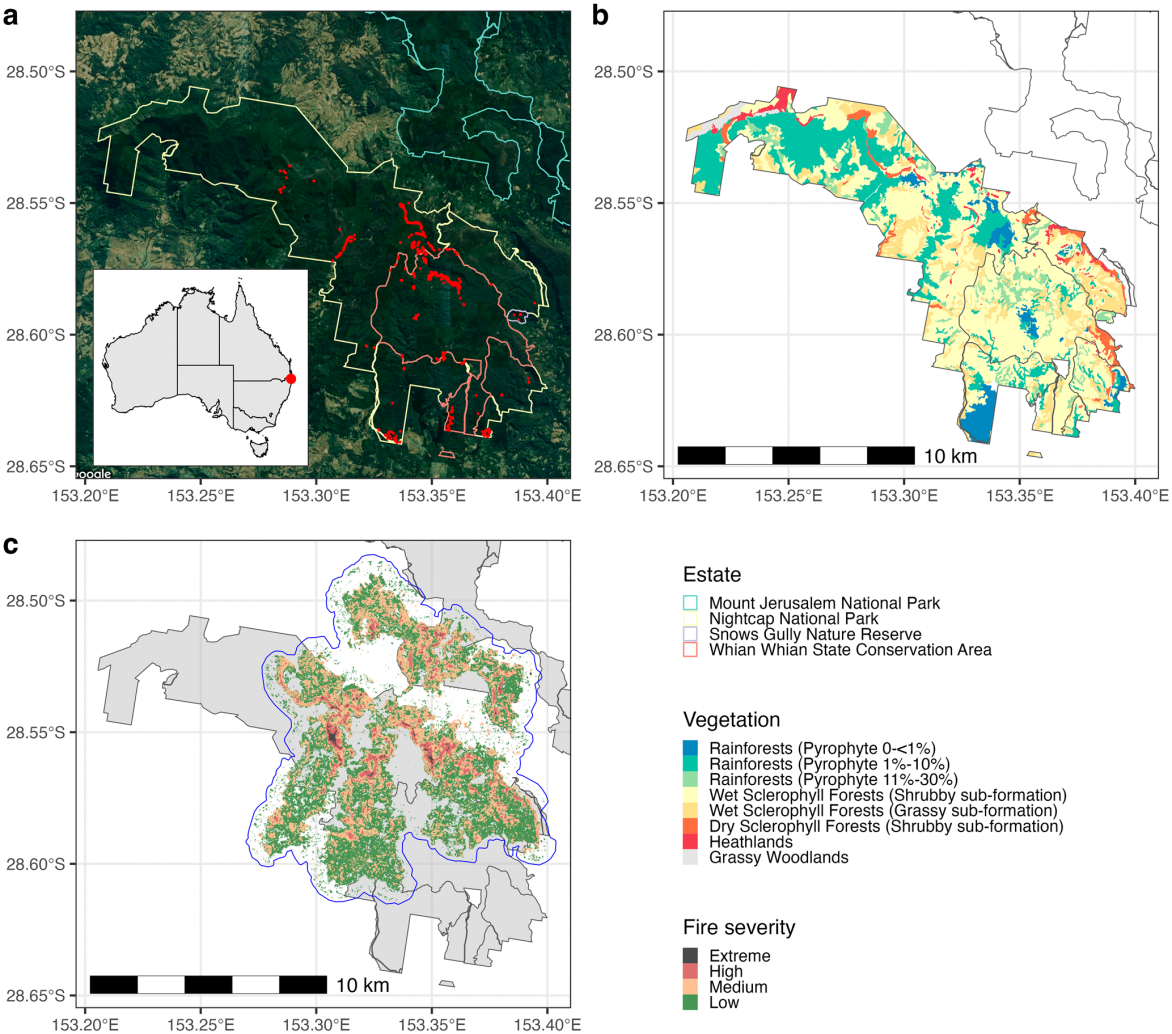
The Nightcap reserves study area. (a) Map of the Nightcap reserve estates, showing the locations of genetic samples from 12 species included in this study (sample points are deidentified). (b) High‐resolution vegetation map of the Nightcap reserves. (c) Fire Extent and Severity Map (FESM v3.0) of the 2019/20 fires across the local region encompassing the Nightcap reserves, illustrating burn intensities classified as low, medium, high, and extreme. The blue outline represents the buffer area used in fire impact calculations.

The Nightcap reserves, comprising Nightcap National Park, Whian Whian State Conservation Area, and Snows Gully Nature Reserve, are located in the Nightcap Range, Northern Rivers, New South Wales (Australia), covering an area of 104 km^2^ (Figure 1a). The 2019/20 bushfires within the Nightcap reserves began with a lightning strike in the Terania Basin on 7 November 2019 and were then driven by north‐westerly winds (40 km/h), low humidity (< 5%), and temperatures up to 38°C (Figure 1b). The fire spread from north to south, moving uphill, with embers igniting adjacent ridges (Peacock and Baker 2022).

### 2.2 Spatial Analysis

#### 2.2.1 Vegetation Mapping

In 2023, the NSW National Parks and Wildlife Service developed a detailed vegetation map for the Nightcap reserves to support fire management and ecological assessments. Vegetation classifications followed Keith's (2004) vegetation formation system. Aerial Photographic Interpretation used high‐resolution Leica Geosystems ADS40 imagery analyzed with Stereo Analyst 3D in ArcGIS to delineate vegetation polygons based on crown structure and color. Fieldwork refined these polygons through 647 rapid site assessments and nine systematic full floristic surveys (20 × 20 m quadrats). Rapid assessments recorded GPS coordinates, dominant species, vegetation type, and notable plants. Full floristic surveys documented vascular species composition, vegetation structure, and Braun‐Blanquet cover‐abundance scores. Findings from both fieldwork methods informed final vegetation classifications.

Vegetation formations were categorized as Rainforest, Wet Sclerophyll (grassy or shrubby), Dry Sclerophyll, Heathlands, or Grassy Woodlands (Keith 2004). Rainforests were defined as areas with ≥ 70% canopy cover from rainforest species (plants that do not require periodic fire for regeneration). Areas with 30% or more canopy cover from pyrophytic species (plants requiring periodic fire for regeneration or germination, such as *Eucalyptus*, *Corymbia*, *Lophostemon*, *Syncarpia*, and *Acacia*) were reclassified as non‐rainforest (key in Appendix 1). Rainforest vegetation was further classified based on the proportion of pyrophytic emergent species in the canopy, with three categories: < 1%, > 1%–10%, and 11%–30% pyrophytic canopy cover.

#### 2.2.2 Fire Extent and Severity

To evaluate the impact of the 2019/20 fires on vegetation in the Nightcap reserves, we analyzed the 2019/20 Fire Extent and Severity Map (FESM v3) dataset (NSW Department of Climate Change, Energy, the Environment and Water 2020) along with the detailed vegetation map. Using QGIS v3.26.1‐Buenos Aires, a buffer zone of 0.001° (~100 m) was applied around the fire‐affected areas to encompass high‐risk zones. This buffer zone was used to trim the vegetation map, limiting the analysis to areas that were burned or at high risk of burning, thereby reducing bias in further calculations.

Burn data were analyzed alongside the vegetation map in RStudio v2023.12.1 + 402 with R v4.3.1 (R Core Team 2021; RStudio Team 2020), using the packages *sf* v1.0–19 (Pebesma 2018), *raster* v3.6–26 (Hijmans et al. 2025b), *terra* v1.7–78 (Hijmans et al. 2025a), and *exactextractr* v0.10.0 (Baston et al. 2023). We quantified the burn extent across each vegetation type within four fire severity classes (low, medium, high, and extreme) and total burn area.

To further interpret the data, we introduced a metric called the “burn area ratio”. This metric compares the observed proportion of burned area for each vegetation type to the proportion expected if all vegetation types had burned uniformly relative to their total area within the study region (Appendix 2). A burn area ratio > 1× indicates that a vegetation type burned disproportionately more than expected, whereas a ratio < 1× suggests that it burned less than expected under the null hypothesis (all vegetation burns equally). This approach allowed us to identify vegetation types that were particularly vulnerable or resilient to fire.

### 2.3 Genomic Analyses

#### 2.3.1 Target Species

To estimate the impact of the fires upon genetic diversity, we analyzed genetic data sampled from a range of local, rainforest‐associated flowering trees (Table 2). This included both rare species endemic to the Nightcap Range, including federally listed Endangered *Uromyrtus australis* A.J. Scott, Critically Endangered *Eidothea hardeniana* P.H. Weston & Kooyman, and Endangered *Elaeocarpus sedentarius* Maynard & Crayn, and nine common species: *Argyrodendron trifoliolatum* F.Muell., *Ceratopetalum apetalum* D.Don, *Diploglottis australis* (G.Don) Radlk., *Doryphora sassafras* Endl., *Hicksbeachia pinnatifolia* F.Muell., *Planchonella australis* (R.Br.) Pierre, *Schizomeria ovata* D.Don, *Sloanea australis* (Benth.) F. Muell., and *Sloanea woollsii* F. Muell. These are all rainforest trees, with heights ranging from 10–15 m (*H. pinnatifolia*, *U. australis* , and *N. dealbata* ) to over 45 m (*A. trifoliolatum* and *S. woollsii*) (Table 1).

**TABLE 1:** Summary of genetic samples analyzed per species within the Nightcap reserves.

Species	Family	Common name	*n*	Loci
*Argyrodendron trifoliolatum* F.Muell.	Malvaceae	White booyong	98	2,216
*Ceratopetalum apetalum* D. Don	Cunoniaceae	Coachwood	83	423
*Diploglottis australis* (G.Don) Radlk.	Sapindaceae	Native tamarind	33	5,697
*Doryphora sassafras* Endl.	Atherospermataceae	Sassafras	52	2,140
☆ *Eidothea hardeniana* P.H.Weston & Kooyman	Proteaceae	Nightcap oak	216	4230
☆ *Elaeocarpus sedentarius* Maynard & Crayn	Elaeocarpaceae	Minyon quandong	75	13,149
*Hicksbeachia pinnatifolia* F.Muell.	Proteaceae	Red bopple nut	29	17,330
*Neolitsea dealbata* (R.Br.) Merr.	Lauraceae	Hairy‐leaved bolly gum	44	3,651
*Schizomeria ovata* D. Don	Cunoniaceae	Crabapple	65	14,814
*Sloanea australis* Benth. & F.Muell.	Elaeocarpaceae	Maiden's blush	43	6,951
*Sloanea woollsii* F.Muell.	Elaeocarpaceae	Yellow carabeen	57	12,874
☆ *Uromyrtus australis* A.J.Scott	Myrtaceae	Peach myrtle	128	7,368

*Note:* The table shows the number of individuals (*n*) and loci per species, after filtering out individuals and loci with high missingness or fixed loci. The endangered target species are indicated (☆).

#### 2.3.2 Sampling and Sequencing

The common species were sampled representatively across their ranges between 2018 and 2023 (Diamon et al. in prep) (Fahey et al. 2024; Yap et al. 2022) and form part of the existing population genetic database for native Australian plants maintained by the Research Centre for Ecosystem Resilience (ReCER) at the Botanic Gardens of Sydney (Rossetto et al. 2019). Here we only analyzed individuals collected within the Nightcap reserves. All known individuals of *Uromyrtus australis*, *Eidothea hardeniana*, and *Elaeocarpus sedentarius* were collected as part of threatened species management strategies (Rossetto et al. 2021).

Approximately 3 g of leaf tissue was sampled from each plant. The samples were stored at −80°C for at least 12 h, then freeze‐dried before being stored in silica gel to ensure preservation until DNA extraction. DArT extracted DNA from ~20 mg of tissue before conducting genotyping using medium‐density DArTseq, a reduced‐representation sequencing method implemented by Diversity Arrays Technology Australia Pty Ltd. (Canberra, ACT, Australia). The DArTseq method involves a genome restriction digest followed by sequencing of the digested products using an Illumina instrument, with genome‐wide single‐nucleotide polymorphisms (SNPs) identified using proprietary analytical pipelines developed by DArT Pty Ltd. (Jaccoud et al. 2001; Kilian et al. 2012).

#### 2.3.3 Diversity Distribution Across Vegetation Types

To assess the distribution of genetic diversity of common and rare species across different vegetation types, we conducted analyses in R (R Core Team 2021). SNP data for each species were filtered using custom scripts (available at: https://github.com/jasongbragg/RRtools). We excluded fixed loci, loci with > 30% missing data, and those with < 96% reproducibility, retaining only one locus per DArT tag. Samples with > 30% missing loci were removed. In total, 923 genetic samples were analyzed across the 12 species (Table 1).

Genetic diversity was assessed by calculating both total and private alleles for each vegetation type, categorized into common alleles (minor allele frequency [MAF] > 5%) and rare alleles (MAF 1%–5%). Generalized linear models (GLMs) were constructed using the *stats* v4.3.1 package to evaluate normalized allele counts (calculated as the proportion of total alleles within each species) for both private and total alleles per vegetation type. Species, vegetation type, and the number of individuals (*n*) were included as predictors to estimate effect sizes and assess statistical significance. Genetic diversity evenness was quantified by calculating the observed heterozygosity of common alleles (MAF > 5%) for each individual species. A GLM was then constructed using vegetation type and species as predictors.

GLMs were chosen for this analysis due to their flexibility in handling varying sample sizes and uneven distributions of individuals across species and vegetation types. They can model different types of response variables and capture nonlinear relationships, making them ideal for assessing the effects of species, vegetation type, and sample size on genetic diversity. GLMs also provide clear estimates of effect sizes and statistical significance, making them well‐suited to quantify and interpret the relationships in this study.

### 2.4 Management Recommendations

To address the question of how to best manage the three threatened species in situ, considering practical constraints, genetic diversity, and future fire risks, we first defined spatial management units and then calculated genetic value and fire risk scores for each unit. The analysis used spatial and genetic data for *Uromyrtus australis, Eidothea hardeniana*, and *Elaeocarpus sedentarius* from known individuals within the Nightcap reserves. All analyses were conducted in RStudio v2023.12.1 + 402 with R v4.3.1 (R Core Team 2021; RStudio Team 2020).

#### 2.4.1 Defining Spatial Management Units

We tested two methods to define management units: a grid‐based method and a cluster‐based method. For the grid‐based method, the study area was divided into grid cells of varying sizes (50, 100, 250, and 500 m). The geographical coordinates (latitude and longitude) of each sample were transformed into the GDA94 coordinate system and assigned to a grid cell. This was done by dividing the coordinates by the grid size, rounding them to the nearest integer grid unit, and adjusting for grid boundaries. Each sample was then grouped into its respective grid cell, represented by a unique management site identifier, to facilitate spatial grouping for subsequent genetic analysis (Figure 4a–d).

The cluster‐based method approached spatial management from a resource‐conscious, spatially aware perspective. In this case, the spatial records of the three target species were clustered simultaneously. The first step applied DBSCAN (Density‐Based Spatial Clustering of Applications with Noise, package *dbscan* v1.1–12) with a maximum distance threshold (epsilon, eps) of 100 m to group spatially close individuals. The DBSCAN algorithm assigned each individual to a cluster or labeled it as noise, with a minimum cluster size of three. These parameters were selected based on their suitability for the data (minPts ≥ dimensions + 1; eps informed by the k‐distance plot's approximate inversion point (Hahsler et al. 2019)) and the management objectives (excluding individuals more than 100 m from the main cluster and avoiding clusters with fewer than 3 individuals, as they are not practical for management).

Density‐based clustering algorithms are particularly effective for grouping spatial distributions of species because they can identify clusters with arbitrary shapes (such as winding tree paths along rivers or ridge lines) and varying sizes or densities, which nondensity‐based algorithms often fail to capture. It is also robust to outliers, making it effective for real‐world spatial data that may contain unusual or erroneous records (Hahsler et al. 2019; Scitovski and Sabo 2020), such as misidentifications or incorrect geodata. Unlike other algorithms that require predefined cluster numbers, DBSCAN uses parameters like distance and minimum points to form clusters, allowing it to efficiently identify dense regions and adapt to diverse cluster patterns.

For clusters with perimeters exceeding a defined value (700 m), iterative *k*‐means clustering (from *stats* v4.3.1) was used to split them into smaller units. This threshold was established based on on‐the‐ground planning, as managers could realistically manage areas with perimeters no larger than 700 m. Differing limits on perimeter or area depending on the goals of the project can easily be substituted at this step. Iterative *k*‐means clustering helps to create compact, well‐separated clusters by minimizing the variance within each group, ensuring that objects within each cluster are as similar as possible (Xu and Wunsch 2005). This process continued iteratively until all clusters had perimeters below the threshold (Figure 4e–g). Overall, this approach emphasizes ecologically meaningful groupings while ensuring the final clusters are manageable from a practical standpoint, thus balancing ecological integrity with management feasibility.

#### 2.4.2 Determining Genetic Value

To calculate a genetic value score for each management unit/site, we focused on three key aspects of genetic EBVs (Hoban et al. 2022): genetic diversity (richness and evenness), genetic differentiation, and inbreeding. We tested several metrics (Appendix 3) and ultimately selected the following: total common alleles per management group (for genetic diversity richness), average heterozygosity per group (for genetic diversity evenness), mean Euclidean distance to other genetic groups (for genetic differentiation), and mean Euclidean distance within management units to define genetically diverse (minimally related) groups. We chose to use total alleles and heterozygosity because they are typical metrics in population genetics associated with the EBVs. The inbreeding coefficient itself (*F*_*IS*_) was excluded, as it is directly correlated with heterozygosity (*F*_*IS*_ = 1–(*H*_*O*_/*H*_*E*_)) when calculated within a species, making its inclusion redundant in this context. Effective population size (*N*_*E*_) was not included in this analysis because it cannot be reliably calculated with our data type; however, it could be a useful metric in this context.

We chose to use Euclidean distances within and between groups rather than kinship or *F*_*ST*_ for several reasons. Kinship tends to provide poor resolution in DArT data for more distant relatives, leading to inaccurate clustering and an unreliable representation of genetic relationships (McMaster et al. 2024). While *F*_*ST*_ is widely used, it is less effective for small sample sizes and may not be meaningful over small spatial scales (Willing et al. 2012), as in our study. *F*_*ST*_ could be more suitable in a regional‐scale study using this framework. On the other hand, Euclidean distances are easier to interpret and apply to datasets of varying size, offering a straightforward measure of genetic divergence based on overall variation between samples (Excoffier et al. 1992; Meirmans and Hedrick 2011). This method is not dependent on biological assumptions, making it applicable to any population, regardless of inbreeding or ploidy (Shirk et al. 2017). Thus, it ensures greater consistency and interpretability, especially when dealing with diverse and complex datasets. Other pairwise distance measures, such as Bray–Curtis or mismatch distance (based on allelic differences), could also be suitable in this context (Shirk et al. 2017). These alternatives more directly account for genetic differences but may yield slightly different results compared to Euclidean distance, depending on the data structure and study objectives.

These diversity metrics were calculated for each species (raw G‐value) and normalized by dividing by the maximum value per species (final G‐value). To determine the raw G‐value for each management unit, we computed a weighted sum of the *n* selected genetic variables, where each variable $v_{i}$ represents a distinct genetic metric (e.g., heterozygosity, allele counts) and $w_{i}$ is its user‐defined weight, reflecting its importance in the analysis. We weighted our variables equally. The formula for the raw G‐value is:

$$G-\text{value}\;\left(\mathrm{raw}\right)=\sum_{i=1}^{n}w_{i}\times v_{i}$$

Next, the G‐value is normalized to ensure comparability across different species or datasets. This normalization is done by dividing the raw G‐value by the maximum raw G‐value observed for any site within the species:

$$G-\text{value}\;\left(\text{final}\right)=\frac{G-\text{value}\;\left(\mathrm{raw}\right)}{\mathrm{Max}\;G-\text{value in species}\;\left(\mathrm{raw}\right)}$$

The resulting final G‐value ranges from 0 to 1, with higher values indicating greater genetic diversity and less inbreeding. For species co‐occurring in the same management unit, the G‐values are calculated separately for each species and can be added together to assess the genetic value of the unit in a multispecies context. For more details, see Appendix 4.

#### 2.4.3 Determining Risk

To incorporate fire risk into our in situ prioritization scheme, we calculated the risk for each management group based on the proportion of overlap with different vegetation types and their associated burn area ratios. Using *sf* v1.0–19, we first performed a spatial intersection between cluster boundaries and vegetation types, calculating the area of overlap. This was used to compute the proportion of overlap for each cluster with each vegetation type. We then summarized the overlap and calculated the total risk by multiplying the proportion of overlap by predefined burn area ratios for each vegetation type. Final risk values were assigned to each cluster based on these calculations and are on the same scale as the original burn area ratios.

## 3 Results

### 3.1 Distribution of Genetic Diversity

**FIGURE 2:**
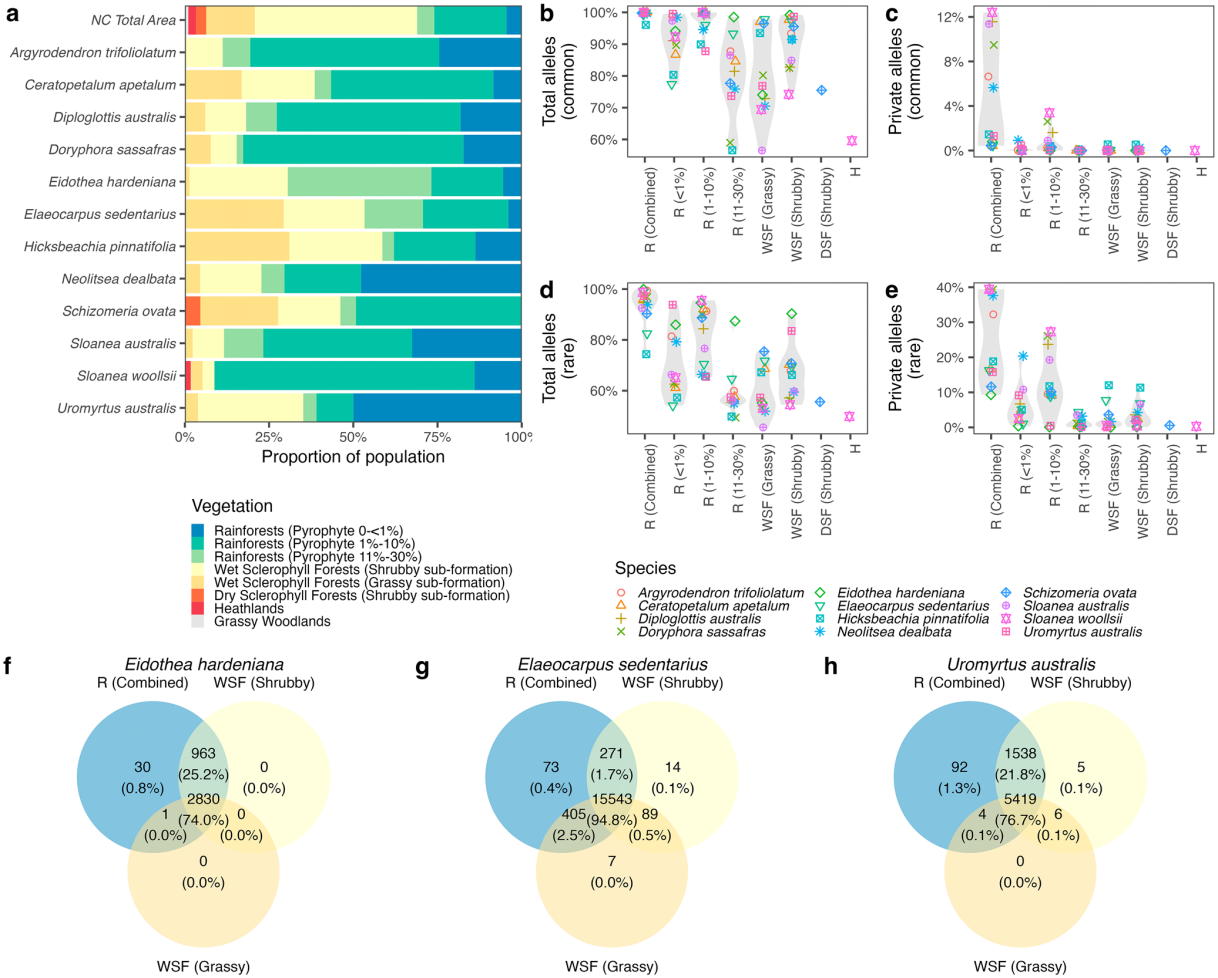
Distribution of 12 target species across vegetation types. (a) Proportion of each species' population distributed across vegetation types. (b and d) Percentage of the total number of common (b) and rare (d) alleles per species found within each vegetation type. (c and e) Percentage of the total number of common (c) and rare (e) alleles per species found that is private to each vegetation type. (f and g) Venn diagrams illustrating the distribution of common alleles across vegetation types for three target endangered species.

The spatial distribution of the 12 species varied across the Nightcap reserves. Most species were found primarily in rainforest vegetation types, though some species had specific habitat preferences. *Eidothea hardeniana* was more common in disturbed rainforest areas that had a higher proportion of pyrophytes. *Elaeocarpus sedentarius* was more frequently found in wet sclerophyll habitats than in other types of vegetation. *Uromyrtus australis* had the largest population in undisturbed rainforest areas, especially those with less than 1% pyrophytic species (Figure 2a).

All 12 species retained over 95% of their common alleles within rainforest vegetation types, with more than 85% located in areas with 1%–10% pyrophytic emergent crown cover (Figure 2h). For the three threatened species, over 99% of their common alleles were found in rainforest habitats (Figure 2f,g), emphasizing the importance of these areas for preserving their genetic diversity.

Common (MAF > 5%) private alleles were scarce across all vegetation types, comprising less than 4% of total alleles for any species. However, up to 12% of common alleles were exclusive to rainforest vegetation (Figure 2c). Rare allele distributions were more variable: while over 70% were found in rainforest habitats (Figure 2d), many were restricted to specific vegetation types (Figure 2e).

Generalized linear models (GLMs) revealed that the number of individuals (*n*) had a significant effect on the total allele counts for both common (MAF > 5%) and rare (MAF 1%–5%) alleles. However, the effect size was minimal (< 0.003), indicating a negligible influence. Rainforest vegetation types (considered both as a combined category [all pyrophyte levels] and individually as 0%–1% pyrophyte cover and 1%–10% pyrophyte cover) had a significant positive impact on the total number of common alleles, with effect sizes estimated at 0.19–0.22. Similarly, total rare alleles were significantly higher in the combined rainforest category and the 1%–10% pyrophyte cover category, with effect sizes ranging from 0.24 to 0.27 (Appendix 5.1–2).

No significant associations were found between species, vegetation type, or number of individuals and common (MAF > 5%) private alleles. In contrast, rare (MAF 1%–5%) private alleles were significantly negatively associated with *Eidothea hardeniana*, suggesting that this species harbors fewer rare private alleles across vegetation types. Rare private alleles were significantly positively associated with rainforest vegetation overall (Appendix 5.3–4).

Individual heterozygosity was not significantly associated with any vegetation type, with none of the vegetation categories showing significant effects on heterozygosity. However, most species had a significant effect on heterozygosity; many species were associated with significantly higher heterozygosity (*Ceratopetalum apetalum*, *Diploglottis australis*, *Doryphora sassafras*, *Eidothea hardeniana*, *Elaeocarpus sedentarius*, *Neolitsea dealbata*, *Sloanea australis*, and *Sloanea woollsii*), while *Uromyrtus australis* exhibited significantly lower heterozygosity (Appendix 5.5). Endangered species did not consistently have lower heterozygosity than more common species, suggesting that heterozygosity levels do not directly correlate with conservation status in this case.

### 3.2 Differences in Fire Severity And Extent

**FIGURE 3:**
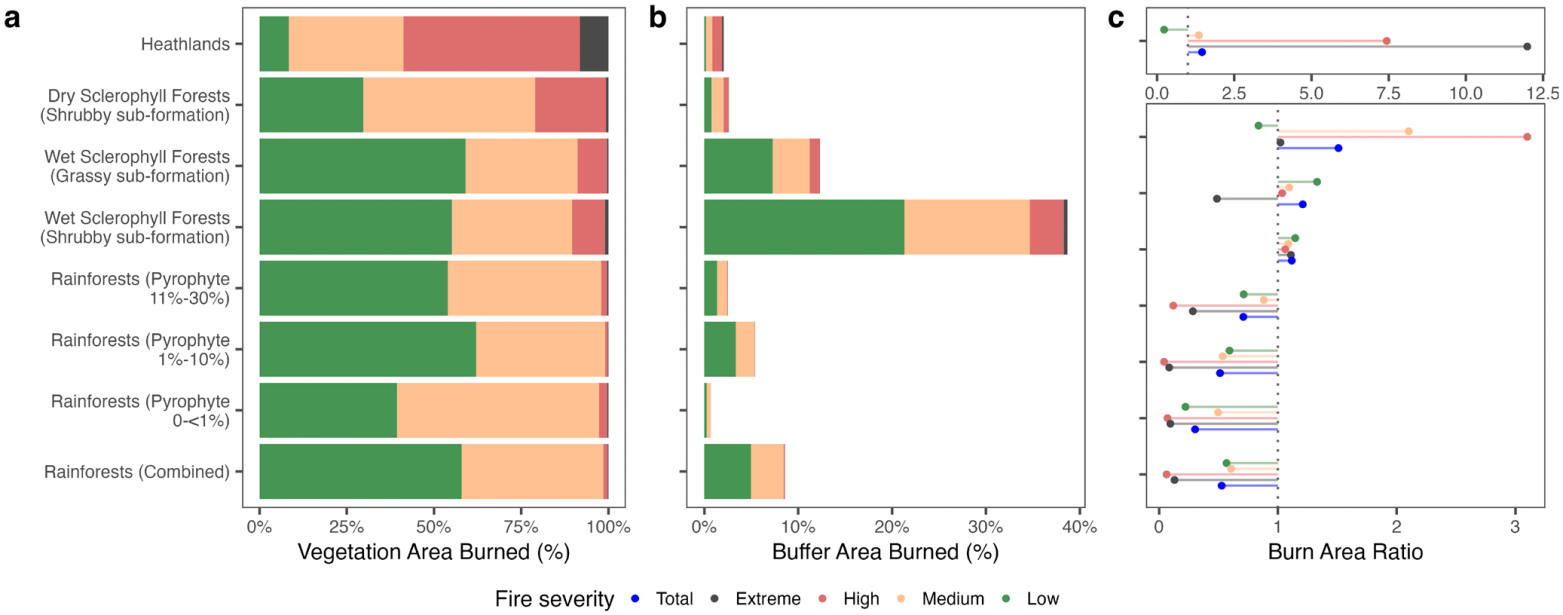
Summary of burn statistics across vegetation types. (a) Proportion of the total burned area per vegetation type, categorized into low, medium, high, and extreme severity classes. (b) Percentage of the buffer zone area burned for each vegetation type. (c) Burn area ratio per vegetation type and severity class. This metric compares the observed proportion of burned area for each vegetation type to the proportion expected if all vegetation types burned uniformly relative to their total area within the study region. A burn area ratio > 1× indicates that a vegetation type burned disproportionately more than expected, whereas a ratio < 1× suggests that it burned less than expected.

The 2019/20 fires in the Nightcap reserves affected different vegetation types to varying degrees. Overall, 36.4% of the Nightcap reserves were burned, with most of the burned area experiencing low intensity (53.7% of the total burn area). Total burn area across vegetation types consistently increased with the presence of pyrophytic emergent species, even within rainforest areas (Table 2 and Figure 3).

**TABLE 2:** Summary of Nightcap vegetation areas and burn metrics.

Vegetation	Nightcap area	Buffer area	Burn area [burn area ratio]				
			Low	Medium	High	Extreme	Total
Rainforests (Pyrophyte 0%–< 1%)	4.75 (4.59%)	2.04 (3.48%)	0.16 [0.22×]	0.23 [0.5×]	0.01 [0.07×]	0.001 [0.09×]	0.4 [0.3×]
Rainforests (Pyrophyte 1%–10%)	22.39 (21.65%)	9.59 (16.37%)	1.96 [0.59×]	1.17 [0.54×]	0.03 [0.04×]	0.01 [0.08×]	3.16 [0.51×]
Rainforests (Pyrophyte 11%–30%)	5.3 (5.13%)	3.22 (5.5%)	0.79 [0.71×]	0.65 [0.88×]	0.02 [0.12×]	0.01 [0.28×]	1.47 [0.71×]
Wet Sclerophyll Forests (Shrubby subformation)	50.37 (48.71%)	31.58 (53.91%)	12.5 [1.15×]	7.81 [1.09×]	2.13 [1.06×]	0.22 [1.11×]	22.66 [1.12×]
Wet Sclerophyll Forests (Grassy subformation)	15.05 (14.55%)	9.28 (15.84%)	4.26 [1.33×]	2.31 [1.1×]	0.61 [1.04×]	0.03 [0.49×]	7.21 [1.21×]
Dry Sclerophyll Forests (Shrubby subformation)	3.13 (3.03%)	1.58 (2.7%)	0.46 [0.84×]	0.75 [2.1×]	0.31 [3.1×]	0.01 [1.02×]	1.53 [1.51×]
Heathlands	2.42 (2.34%)	1.29 (2.2%)	0.1 [0.23×]	0.4 [1.35×]	0.61 [7.44×]	0.1 [11.99×]	1.21 [1.46×]
Overall	103.41 (100%)	58.58 (100%)	20.23	13.31	3.72	0.37	37.64

*Note:* Unbracketed values represent area in square kilometers (km^2^). Values in round brackets () indicate the percentage of the total area within the specified zone (either Nightcap reserves or buffer zone). Burn areas (km^2^) and burn area ratios are shown in square brackets []. The burn area ratio compares the observed burn area to the expected burn area under uniform burn distribution. A ratio of 1× indicates the burn area is as expected; < 1× means the burn area is smaller than expected, and > 1× means it is larger than expected. For example, a ratio of 2× means the observed burn area is twice as large as expected, while a ratio of 0.5× means the burn area is half the expected area. Data are categorized by vegetation type and burn severity.

Rainforest vegetation exhibited lower‐than‐expected burn areas across all burn types, with burn area ratios consistently less than 1.0×. However, the total burn area ratio increased as the presence of pyrophytic species in the canopy increased. Wet sclerophyll forests experienced higher total burn areas than rainforests, with grassy subformations proving marginally more flammable than shrubby subformations, exhibiting burn area ratios of 1.12× and 1.21×, respectively (Table 2). The wet sclerophyll shrubby subformation, which comprises 48.71% of the reserves, contributed the largest total burn area, totaling 22.66 km^2^ (Figure 3).

Dry sclerophyll forests were more flammable, with higher burn area ratios, particularly for medium (2.1×) and high (3.1×) intensity burns. Heathlands, despite covering only 2.31% of the reserve area, experienced notably higher burn intensity, with high burn areas (7.44×) and extreme burn areas (11.99×) considerably exceeding expectations (Figure 3c).

### 3.3 Management Unit Outcomes

**FIGURE 4:**
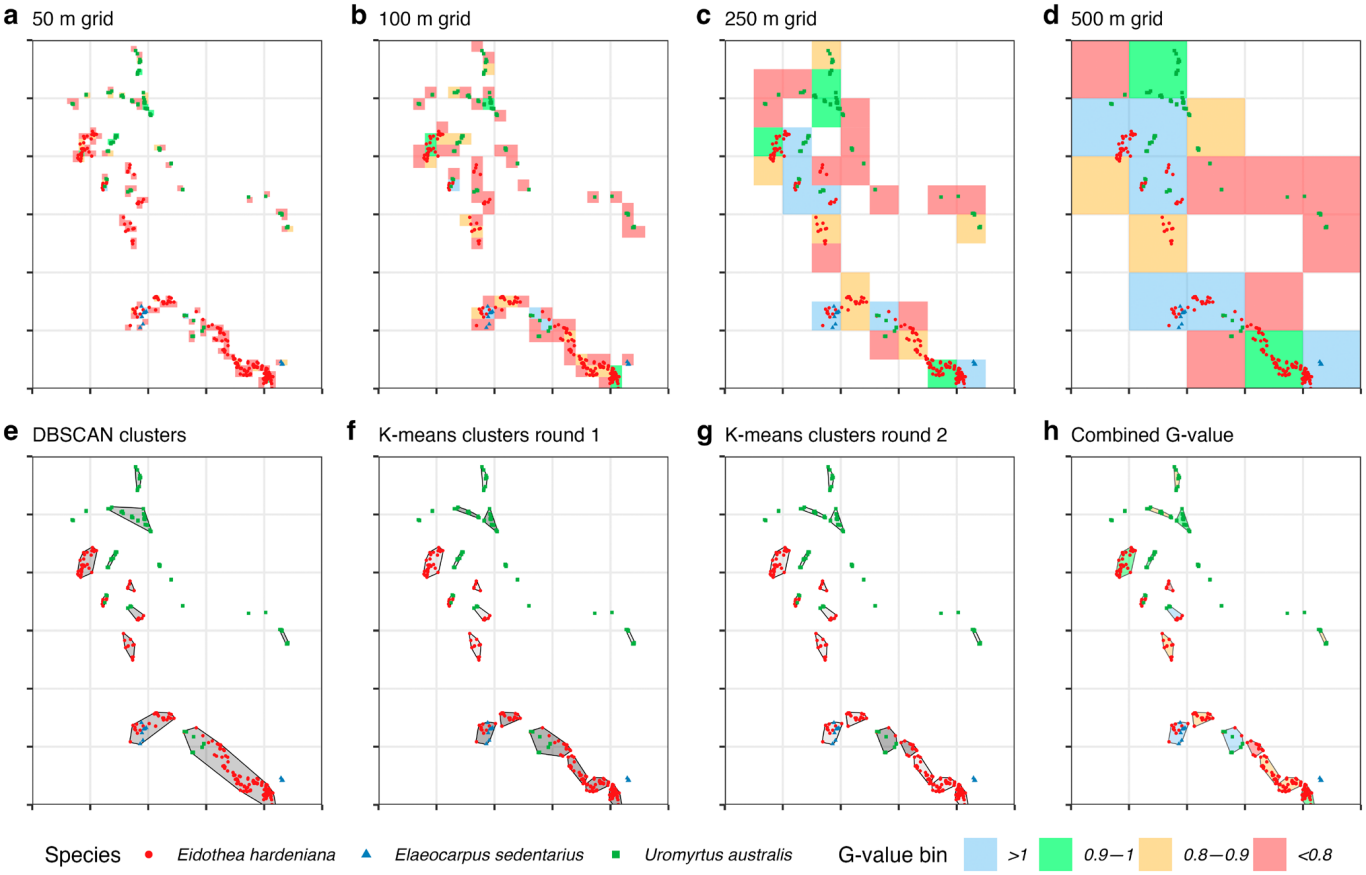
Comparison of the grid method and clustering algorithm for identifying management units. The area shown is a subset of the Nightcap reserves, encompassing individuals of three target endangered species. Latitude and longitude identifiers have been removed. Axis ticks are 500 m apart. (a–d) Application of the grid method across the area with increasing grid cell sizes. Fill color indicates the G‐value, a normalized weighted sum of genetic diversity metrics. The grid method proved ineffective in identifying ecologically relevant or management‐appropriate units. (e–g) Stages of the iterative clustering algorithm. (e) Initial clustering using DBSCAN, which identifies density‐based clusters. (f and g) Subsequent steps split DBSCAN clusters with perimeters exceeding 700 m into smaller units until all clusters meet the perimeter threshold. (h) Final clusters produced by the algorithm, with fill color indicating the G‐value. These clusters are ecologically meaningful (representing closely clustered individuals) and meet management requirements.

The grid‐based and cluster‐based methods produced different results for defining management units. The grid‐based method resulted in groups with low genetic diversity at smaller grid cell sizes (50–100 m) and overly large, impractical areas at larger grid cell sizes. It also failed to capture species distributions accurately, often splitting adjacent groups arbitrarily (Figure 4a–d). The clustering method, with applied limitations such as perimeter size and distance thresholds to account for on‐ground logistics, produced spatially reasonable and manageable groups (Figure 4e–h). As a result, we used only the cluster‐based management units in our subsequent risk and G‐value assessments.

### 3.4 G‐value vs. Risk Optimization

**FIGURE 5:**
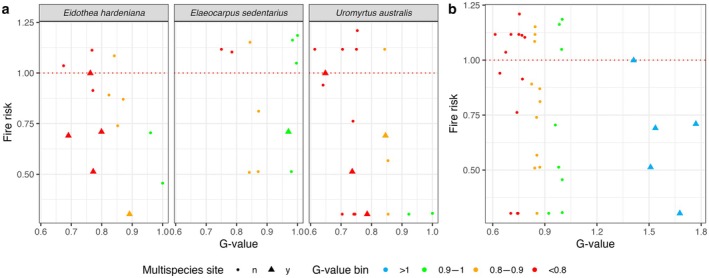
G‐value and fire risk scores of management sites. The G‐value reflects an additive metric incorporating the proportion of total alleles, heterozygosity, genetic distance within and between sites, and the number of individuals. Fire risk is determined by the total burn area ratio of vegetation types within the site. (a) G‐value and fire risk scores for the three target threatened species individually. (b) Combined G‐value for multispecies sites, calculated as the sum of individual species' G‐values.

The relationship between genetic value (G‐value) and fire risk varied across species. For all three endangered species, the overall fire risk was relatively low (~0.25–1.25×; Figure 5), because most individuals were predominantly located in rainforest areas, which are less fire‐prone (Figure 3). However, there was considerable variation in both G‐value scores and fire risk among the different management units.

For *E. hardeniana* and *U. australis* , the management units exhibited a broad range of G‐values (0.6–1.0). The highest G‐value units (> 0.9) were in low‐risk areas, where fire risk was below 0.75×. In contrast, units with lower G‐values were typically situated in areas with higher fire risk. For *E. sedentarius*, higher G‐value units were more common, with many exceeding a G‐value of 0.9. However, some of these high‐G‐value units were found in areas with elevated fire risk, exceeding a risk level of 1.0 × .

For multispecies management units, where two species co‐occurred, the average G‐value of each species was generally lower than that for single‐species units. These multispecies units were predominantly located in areas with lower fire risk, with most units exhibiting a fire risk of 1.0× or lower.

## 4 Discussion

**FIGURE 6:**
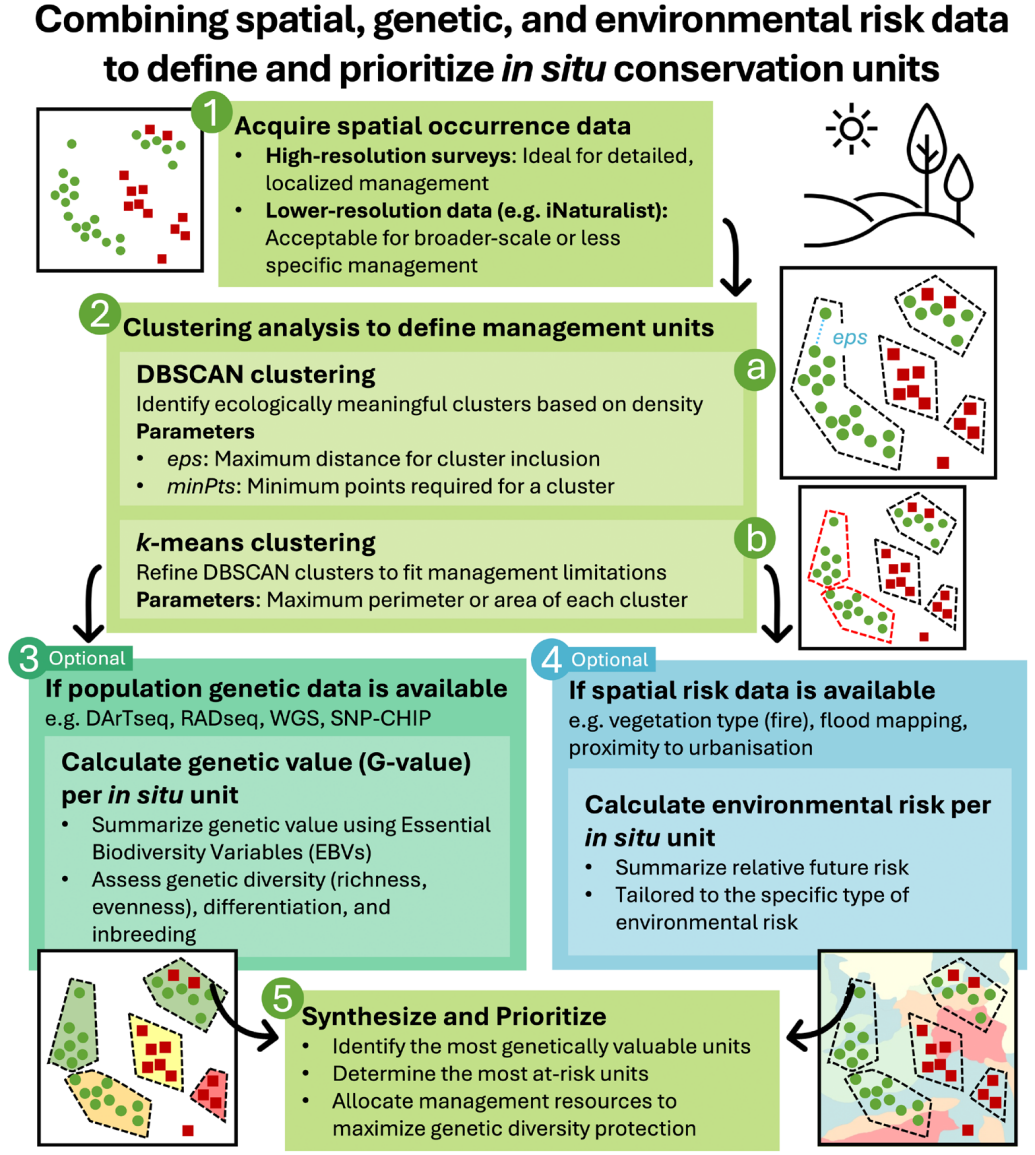
Framework for defining and prioritizing in situ conservation units by integrating spatial, genetic, and environmental risk data. This workflow provides a step‐by‐step guide on how to apply the method, as described in the paper's methods section. It outlines key actions at each stage, highlighting points where methodological choices (e.g., data types, parameters) can be adjusted. The diagrams adjacent to the text boxes visually represent the processes occurring at each step.

In this study, we have integrated population genetic data, remote sensing, and vegetation mapping with an approach that accounts for practical constraints to guide in situ conservation management (Figure 6). This multidimensional framework allowed us to evaluate both the relative fire risk and genetic value of three Endangered or Critically Endangered plant species simultaneously. By doing so, we identified actionable strategies for the efficient allocation of fire‐mitigation resources in the field. Here we discuss the applicability and adaptability of our methods to other contexts and explore the specific implications of our findings for the conservation of our target species and rainforest ecosystems more broadly.

### 4.1 Effectiveness of Clustering Algorithms For Site Selection

A key innovation of this study was the approach to in situ management unit delimitation. Initially we trialed a grid‐based system to identify diversity hotspots, as has been applied in previous studies to identify conservation reserve candidates (Watts et al. 2009), species diversity hotspots (Andrello et al. 2022; Burgess et al. 2019; Gaston 2000; Pacicco et al. 2018; Thuiller et al. 2006), and genetic diversity hotspots (Gugerli et al. 2008). However, we encountered issues with the grid system, which imposes arbitrary cutoffs that fail to reflect ecological boundaries. The limitations of grid‐type analyses in describing ecologically relevant units have been pointed out previously, particularly at smaller scales (Gaston et al. 1998; Marsh et al. 2023).

Interpolation models are also often used for identifying genetic diversity hotspots over large areas (Canestrelli et al. 2010; Chiocchio et al. 2024; Yu et al. 2019); however, we chose not to pursue this approach here because we wanted to prioritize spatially explicit management units as the first step of the process. This also allows us to calculate specific within‐group and between‐group genetic parameters that cannot be done on a continuous scale.

Our approach of using clustering algorithms to define management units proved both practical and effective, particularly in capturing ecologically meaningful groupings. Clustering algorithms have been extensively applied across biological sciences for problems such as gene expression analysis, trait clustering (Kiselev et al. 2017; Ma et al. 2006; Mizikovsky et al. 2022), population structure inference (Pritchard et al. 2000), and genome assembly (Newell et al. 2024). However, their application in spatial ecological contexts remains relatively rare. In contrast, clustering algorithms have seen broader use in nonbiological spatial contexts, such as urban planning and large‐scale regional analyses (Fleischmann and Arribas‐Bel 2022; Kii et al. 2023; Wang et al. 2024). These applications often focus on large‐scale patterns rather than the fine‐scale ecological insights required for conservation planning.

Although some studies have highlighted the value of clustering algorithms in spatial ecological research, particularly for identifying biodiversity hotspots (Lavigne et al. 2010; Nageswara Rao et al. 2007; Pang et al. 2023), their potential for defining targeted, spatially relevant management units for in situ conservation has been underexplored. Density‐based clustering algorithms, in particular, offer notable advantages in identifying meaningful groupings based on proximity and density thresholds, making them highly suitable for conservation applications. By leveraging these methods, we provide a framework for defining actionable management units that align with ecological and practical considerations, addressing a critical gap in spatial applications of clustering algorithms for conservation planning.

This method is highly adaptable for various in situ management scenarios. In our case, sites were limited to a 700 m perimeter, reflecting the practical management range for actions like sprinkler placement, backburning, litter clearance, and firefighter intervention during wildfires. However, different perimeter or area limits can be easily adjusted based on project goals. The only requirement for applying this method is spatial occurrence data for the species of interest, which are increasingly available at large scales through citizen science resources like the Atlas of Living Australia (Belbin et al. 2021), iNaturalist (Mesaglio and Callaghan 2021), and ebird (Sullivan et al. 2014). The method can be scaled up for applications such as identifying large management area candidates across a state or continent, or for smaller projects like this one. The DBSCAN step identifies ecologically meaningful units that are robust to data inaccuracies, such as outliers and incorrect geospatial information. Additionally, its flexibility to incorporate area and perimeter constraints makes it highly adaptable for diverse management tasks, including wildlife fence planning and land buyback programs.

This clustering method is most appropriate for species with thorough occurrence data, such as sysmatic surveys. It is ideal for extensively studied plant species, where most individuals' locations are known. It can also be applied to immobile organisms like corals, or even mobile species, provided that the data are comprehensive (capturing high frequency visited areas). Sparse or biased data can limit the method's effectiveness, as DBSCAN relies on density to detect clusters. Under sampled but biologically important areas may be overlooked if they lack sufficient data points to form a cluster. Researchers must be aware of potential observation biases, particularly in citizen science projects, where areas with higher human activity tend to have more observations. To maximize effectiveness, a systematically surveyed study area is recommended. When properly applied, this method facilitates the identification of ecologically meaningful clusters, enabling managers to take actionable steps.

For further guidance and practical examples, refer to the implementation on GitHub [https://github.com/eilishmcmaster/insitu_workflow]. This repository includes sample data and detailed instructions for replicating the clustering process for a subset of the data presented here, as well as an additional large‐scale example based on koala ( *Phascolarctos cinereus* [Goldfuss, 1817]) records from ALA using area rather than perimeter constraints (Appendix 6). A generalized workflow is also presented in Figure 6.

### 4.2 Risk vs. Reward

Conservation strategies must carefully balance risk and reward, particularly when deciding where and how to apply protections or interventions (Andrello et al. 2022). This study demonstrates the utility of integrating genetic diversity metrics with environmental risk assessments to prioritize conservation actions more effectively.

The “G‐value” index, combining aspects of genetic richness, evenness, differentiation, and inbreeding, offers a practical tool for consolidating genetic data into a single prioritization measure. The weighted approach allows conservation practitioners to prioritize specific genetic attributes, such as total allele capture, heterozygosity, or unique diversity, tailored to the goals of a given project. Composite indices such as the G‐value are widely used across disciplines to guide decision‐making under complex conditions. Examples include the Human Development Index (Ranis et al. 2006), air quality indices (Kanchan et al. 2015), polygenic risk scores (Maamari et al. 2025), and genetic composite traits employed in animal and plant breeding programs (Cameron 1997; Céron‐Rojas and Crossa 2018; Rahimi and Debnath 2023). Even the IUCN Red List categories can be seen as a composite index, integrating multiple criteria to assess extinction risk (IUCN Species Survival Commission 2024). While composite measures are not as directly interpretable as individual genetic measures, they enable more systematic evaluations and comparisons. In this study, conservation units with the highest G‐values represented populations excelling across multiple genetic metrics, demonstrating the index's effectiveness in prioritization.

Another key aspect of this framework is the integration of environmental risk assessments, in this case wildfire, into the conservation framework. Prioritizing in situ management solely based on genetic value may neglect populations at higher risk of disturbance, whereas focusing exclusively on risk can overlook genetically important populations. By combining genetic value with risk evaluations, practitioners are able to address both immediate threats and long‐term resilience of species in situ.

Risk assessments are useful in this context even when they are not fully predictive of future events. In the case of wildfires, while vegetation type can provide a relative measure of risk, dynamic and random factors, such as weather conditions and ignition points, remain unpredictable (Andrews 2018; Murphy et al. 2013; Tedim et al. 2018). The simplification of these complex and dynamic risk factors in an assessment like this will inevitably have limitations, but it still provides valuable insights within a prioritization framework by identifying areas of higher risk. This helps to guide conservation efforts and resource allocation more effectively than ignoring these factors altogether.

This approach for assessing the relative risks posed by diverse environmental factors can be applied to a variety of other contexts, such as mapping drought conditions, flood‐prone areas, evaluating proximity to urbanization, and analyzing agricultural encroachment. For example, meteorological drought prediction maps could be used in a similar manner to identify conservation sites at high risk (Park et al. 2019), as was done with the vegetation map in this study. In the same way, the proximity to urban or agricultural areas contributes to biodiversity risks, such as habitat fragmentation, pollution, and disruptions to ecosystems, which ultimately reduce plant and animal diversity (Campos et al. 2024; Clucas et al. 2018; Gaston 2010). This can serve as an indicator to identify more vulnerable conservation sites. While this approach is effective for evaluating spatially variable risks, it may be less applicable in areas with uniform environmental factors or where microhabitats have a more significant role than broad spatial patterns. Incorporating environmental risk assessments is critical for effective conservation planning (Game et al. 2013; Wilson et al. 2005), and integrating spatial risk evaluations further enhances in situ conservation efforts.

### 4.3 Fire and Rainforest Diversity

Previous studies have shown how recurrent or temporal disturbances affect species diversity across large regional scales (e.g., Nightcap vs. Washpool reserves) (Kooyman et al. 2011; Rossetto et al. 2015), but this study highlights that such disturbances can also impact genetic diversity at much finer spatial scales and shorter time frames. This focus on gene‐level effects reveals critical insights into the resilience of rainforest ecosystems across varying scales. Rainforests are often recognized as being part of environmentally stable refugial areas that support high species diversity (Das et al. 2019; Koenen et al. 2015; Rossetto and Kooyman 2021; Wright 2002). In the case of rainforest‐associated trees within the Nightcap reserves, rainforest vegetation not only harbors a greater number of species but also fosters significantly higher genetic diversity within both common and rare species. Specifically, we found that allelic richness, which is essential for adaptive potential and overall resilience, is notably higher in rainforest habitats with less than 10% pyrophytic species, compared with other vegetation types.

This observed effect is not merely a result of higher individual abundance in rainforest vegetation. While these areas do host more individuals which significantly increase allelic richness, the vegetation types are also independently associated with a significantly greater number of total alleles, both for common and rare alleles, across all 12 species examined. A particularly striking finding is the distribution of common alleles—over 95% of common alleles in all species were found in rainforest vegetation, with up to 12% of these alleles exclusive to rainforest vegetation. In the threatened small‐range species *U. australis* , *E. hardeniana* , and *E. sedentarius*, over 99% of all common alleles were found in rainforest vegetation.

The distributions of common alleles suggest that rainforest habitats offer more than just physical space for species; they provide a range of environmental factors that promote higher genetic variation. The encroachment of pyrophytic species into rainforest vegetation and their associated susceptibility to fire may contribute to the observed differences in genetic diversity among vegetation types. Although many rainforest trees are capable of resprouting after fire, they are not specifically adapted to frequent fire regimes and exhibit varying capacities to tolerate fire (Baker et al. 2022; Benwell 2024; Rossetto and Kooyman 2005).

Our analysis of fire extent and severity across Nightcap vegetation types revealed that an increasing presence of pyrophytic species within the canopy increases the overall flammability of typically fire‐suppressant rainforest vegetation (Baker et al. 2021). This dynamic aligns with the “interval squeeze” model (Enright et al. 2015) whereby the more frequent fires in pyrophyte‐invaded rainforest areas reduce recruitment, growth, and survival of fire‐susceptible plants (Le Breton et al. 2022). Over time, this limits the accumulation of new alleles (e.g., via mutation) and reduces genetic diversity in disturbed areas. Furthermore, increased fire frequency can eliminate older, long‐lived trees—critical reservoirs of genetic variation—compounding the effects of interval squeeze. For the 12 species in our study, many of which are capable of surviving for hundreds or thousands of years (Floyd, 1990), such losses threaten the temporal maintenance of alleles and the resilience of rainforest tree populations, and may account for the significantly higher maintenance of allelic richness in rainforest areas with less pyrophytic canopy cover. These areas act as a rainforest microrefugia, safeguarding and maintaining high concentrations of genetic diversity for both common and threatened species. Given these risks, targeted management interventions would be beneficial to maintain and restore rainforest habitats. Fire planning should prioritize the active protection of rainforests during wildfires, while post‐fire management efforts should focus on reducing the encroachment of pyrophytic species through assisted regeneration. These strategies can help mitigate the loss of genetic diversity and support the long‐term resilience of rainforest ecosystems.

We see this excess diversity in less fire‐prone rainforest vegetation directly in the management assessment, where the highest‐value units typically had lower fire risk than less genetically valuable sites. This demonstrates that, beyond specific in situ management actions, the overall role of rainforest vegetation in maintaining genetic diversity is critical. It is not just the individual conservation units that matter, but the broader protection of low‐fire‐risk rainforest areas.

## 5 Conclusion

By combining innovative tools with practical methodologies, we present a comprehensive and adaptable framework that enhances conservation efforts in the face of increasingly dynamic and risky environments (Figure 6). The integration of genetic, ecological, environmental, and spatial data demonstrates the power of multidisciplinary approaches in solving complex multispecies conservation challenges. The clustering method that we used offers a replicable and flexible model for defining in situ management units, particularly in cases with a clear understanding of species occurrence, sympatric species, or operational constraints. Tools like the G‐value allow for generalized characterization of genetic diversity, supporting more informed decision‐making. Examples of how to apply all steps of our workflow (spatial clustering, G‐value calculation, and risk calculation) can be found here [https://github.com/eilishmcmaster/insitu_workflow]. This workflow can benefit anyone working with spatial data to create management sites, whether or not genetic or environmental risk data (e.g., wildfire, drought, and urbanization) are available.

## Author Contributions

**Eilish S. McMaster:** conceptualization (lead), data curation (equal), formal analysis (lead), investigation (lead), methodology (lead), visualization (lead), writing – original draft (lead). **Richard J. Dimon:** data curation (equal), writing – review and editing (supporting). **Andrew G. Baker:** data curation (equal), investigation (equal), writing – review and editing (supporting). **Craig Harre:** data curation (equal), investigation (equal). **Justin Mallee:** conceptualization (supporting), writing – review and editing (supporting). **Aleks Maric:** data curation (equal), investigation (equal). **Peter Richards:** data curation (equal), investigation (equal). **Matthew Wiseman:** data curation (equal), investigation (equal), writing – review and editing (supporting). **Simon Y. W. Ho:** supervision (equal), writing – review and editing (equal). **Maurizio Rossetto:** funding acquisition (equal), resources (equal), supervision (equal), writing – original draft (supporting), writing – review and editing (supporting).

## Conflicts of Interest

The authors declare no conflicts of interest.

## Supporting information


Data S1.

## Data Availability

The genomic data supporting these analyses is available on Dryad at https://doi.org/10.5061/dryad.nzs7h452p.
