# Supplementary material for: Combining Spatial, Genetic, and Environmental Risk Data to Define and Prioritize In Situ Conservation Units
Source: Ecol Evol. 2025 May 26;15(5):e71251. doi: 10.1002/ece3.71251 (PMC12105916; doi:10.1002/ece3.71251)
Supplement: Supplementary file 1 — Data S1. [file ECE3-15-e71251-s001.docx]

Combining spatial, genetic, and environmental risk data to define and prioritize *in situ* conservation units: Appendices

**CONTENTS**

[1 Vegetation classification 2](#_Toc188517472)

[2 Burn area ratio calculation 4](#_Toc188517473)

[3 Rationale for selected variables in the G-value metric 5](#_Toc188517474)

[4 G-value calculation 7](#_Toc188517475)

[4.1 How the G-value is calculated 7](#_Toc188517476)

[4.2 Variables we used in G-value calculation 7](#_Toc188517477)

[4.2.1 Example of how Euclidean distances were calculated 8](#_Toc188517478)

[4.2.2 Example of how G-value was calculated 9](#_Toc188517479)

[5 Generalized linear model results 10](#_Toc188517480)

[5.1 Total common alleles 11](#_Toc188517481)

[5.2 Total rare alleles 11](#_Toc188517482)

[5.3 Private common alleles 12](#_Toc188517483)

[5.4 Private rare alleles 12](#_Toc188517484)

[5.5 Heterozygosity 13](#_Toc188517485)

[6 Koala example 14](#_Toc188517486)

# Vegetation classification

Key to vegetation types in New South Wales based on Keith (2004) used for classification in the vegetation mapping. Modifications by Baker (2021) are included for wet and dry sclerophyll forests (Baker, 2021). Sub-formations are included where appropriate.

| 1 | Vegetation dominated by trees. | 2 |
| --- | --- | --- |
|  | Trees absent or only as scattered individuals. | 11 |
| 2 | Forests or woodlands dominated by eucalypts. | 3 |
|  | Forests or woodlands where eucalypts are not the main species but may be scattered. | 7 |
| 3 | Tall forests with dominant tree habit tall (typically >30 m) straight trunked eucalypts usually branching at more than half of their height. Understorey usually featuring soft-leaved shrubs, ferns, or herbs, located on fertile coastal soils with >900 mm of annual rainfall. Excludes riverine forests west of the Great Dividing Range. Rainforest species can constitute <70% of crown cover. | **Wet sclerophyll forests**  Shrubby: Understorey dominated by soft-leaved shrubs, sparse grass cover.  Grassy: Understorey dominated by grasses and herbs, sparse shrub cover. |
|  | Forests or woodlands with short to moderate-sized trees (rarely >35 m), lacking ferns and soft-leaved shrubs in the understorey but may include grasses, hard-leaved shrubs, or ephemeral herbs. Found both east and west of the Great Dividing Range. | 4 |
| 4 | Understorey with plants tolerant of waterlogging (sedges, rushes, reeds), found in damp low-lying areas or near rivers, lakes, and swamps. | **Forested wetlands** |
|  | Understorey typically lacking plants that tolerate waterlogging or inundation. | 5 |
| 5 | Forests or woodlands with hard-leaved shrubs, rarely dominated by 'box' eucalypts. Dominant tree habit is short to moderately tall trees (rarely >35 m), usually branching at less than half of their height. Eucalypt trunks slightly crooked or leaning. Understorey generally lacks ferns and shrubs with broad soft leaves, but may include abundant grasses, hard-leaved shrubs or ephemeral herbs.  Found on infertile soils along the coast, tablelands, and western slopes with >500 mm annual rainfall. | Dry sclerophyll forests  Shrubby: understoreys dominated by soft-leaved shrubs but only sparse grass cover.  Grassy: understoreys dominated by a more continuous cover of grasses and herbs but only sparse shrub cover. |
|  | Woodlands or forests with few hard-leaved shrubs, often with 'box' eucalypts, grasses prominent except in semi-arid areas. Found on various soils west of the Great Dividing Range, and fertile soils on coast, tablelands, and western slopes. | 6 |
| 6 | Woodlands or forests 15–35 m tall with ground cover of perennial tussock grasses, herbs, few ephemeral plants, and sparse shrubs. Found on fertile soils in coastal lowlands, tablelands, and western slopes with >500 mm annual rainfall. | **Grassy woodlands** |
|  | Woodlands or open woodlands 5–20 m tall, with ground cover of ephemeral herbs and grasses following rain, perennial tussock grasses, and drought-tolerant shrubs, including chenopods. Found on various soils in western plains with <500 mm annual rainfall. | **Semi-arid woodlands**  Grassy: Floodplain areas, often dominated by eucalypts >15 m, understorey of grasses and/or chenopods.  Shrubby: Peneplains and hills, eucalypts rarely >15 m, open understorey with drought-tolerant shrubs, variable grasses. |
| 7 | Forests with closed canopies and trees with soft leaves. Mainly found on the coast and escarpment with >1000 mm annual rainfall, and in dry rocky gorges and north-western slopes. | **8** |
|  | Woodlands and open forests with open canopies, typically featuring wattles, casuarinas, or paperbarks with hard, pendulous leaves. Common on western plains, with limited occurrences on the coast and tablelands. | **10** |
| 8 | Trees that tolerate tidal inundation with a sparse or absent understorey. Located in tidal estuaries along the coast. | **Saline wetlands** |
|  | Trees not tolerant of tidal inundation, with an understorey ranging from open to dense. Found on the coast, escarpment, and north-western slopes, but not in tidal estuaries. | **9** |
| 9 | Trees from various families with broad, soft leaves, often accompanied by vines. The understorey includes ferns and herbs. Located in coastal lowlands, islands, and escarpments on fertile soils, with limited occurrences on the north-western slopes. Rainforest species must constitute >70% of crown cover. | **Rainforests** |
|  | Canopy dominated by wattles with fine, feathery leaves, and a sparse understorey of shrubs and sedges. Found only in steep, rocky foothills and gorges on the south coast and ranges. | **Dry sclerophyll forests** |
| 10 | Open forests 15–30 m tall with plants tolerant of periodic inundation or waterlogging. Dominant trees include casuarinas or paperbarks. The understorey features sedges, rushes, or grasses, but no chenopods. Found only on the coast and tablelands near streams, lakes, and swamps. | **Forested wetlands** |
|  | Woodlands or open woodlands 5–20 m tall with canopies that rarely touch, generally lacking plants tolerant of inundation or waterlogging. Dominant trees include wattles or casuarinas. The understorey consists of grasses, herbs, and drought-tolerant shrubs, including chenopods. Found on western plains with <500 mm annual rainfall. | **Semi-arid Woodlands**  **Grassy: Floodplains, trees >15 m, with grasses and/or chenopods in the understorey.**  **Shrubby: Peneplains and hills, trees <15 m, with drought-tolerant shrubs and variable grasses.** |
| 11 | Vegetation dominated by plants tolerant of prolonged seasonal burial in snow. Restricted to the alpine zone of the southern tableland (> 1800 m elevation). | **Alpine complex** |
|  | Vegetation dominated by plants that cannot tolerate prolonged seasonal burial in snow. Distributed in non-alpine landscapes (< 1800 m elevation). | **12** |
| 12 | Vegetation with plants tolerant of periodic inundation or waterlogging, dominated by sedges, rushes, reeds, grasses, or succulent herbs, sometimes submerged or floating aquatic herbs. Soils deep, often black or dark grey with organic matter. | **13** |
|  | Vegetation with few if any plants tolerant of periodic inundation or waterlogging, usually dominated by shrubs or grasses, sometimes sedges, never submerged or floating aquatic herbs. Soils may vary in colour and moisture. | **14** |
| 13 | Dominated by shrubs, sedges, grasses, or non-succulent herbs that tolerate freshwater inundation or waterlogging. Found in swamps with humic or gleyed soils on the coast, tablelands, western slopes, and plains. | **Freshwater wetlands** |
|  | Dominated by herbs (including succulents), grasses, or rarely shrubs tolerant of saline water inundation or waterlogging. Found in tidal estuaries on the coast, and salt lakes on the western plains. | **Saline wetlands** |
| 14 | Vegetation dominated by perennial tussock grasses with herbs, very rare shrubs. Generally found on clay soils in flat to undulating terrain on coast, tablelands, western slopes, and plains. | **Grasslands** |
|  | Vegetation dominated by shrubs, with occasional or absent perennial tussock grasses. Generally found on sandy or loamy soils of coast, tablelands, and western plains. | **15** |
| 15 | Vegetation dominated by hard-leaved but not drought-tolerant shrubs, usually with perennial sedges, herbs, and grasses, few ephemeral plants. Restricted to infertile soils in exposed sites along coast and tablelands with >800 mm annual rainfall. | **Heathlands** |
|  | Vegetation dominated by drought-tolerant shrubs, including chenopods, with some perennial grasses and herbs, abundant ephemeral grasses and herbs after rain. Widespread on various soils in western plains with <500 mm annual rainfall. | **Arid shrublands** |

# Burn area ratio calculation

The burn area ratio compares the observed proportion of burned area for each vegetation type to the proportion expected if all vegetation types had burned uniformly, based on their total area within the study region. A burn area ratio greater than 1 indicates that a vegetation type burned more than expected, while a ratio less than 1 suggests it burned less than expected under the null hypothesis (all vegetation burns equally). This method helps to identify vegetation types that are particularly vulnerable or resilient to fire.

Example calculation:

- The heathland vegetation type covers 1.29 km² of the buffer zone, and 1.21 km² of it burned.
- In total, 37.64 km² of the buffer zone burned, and the buffer zone is 58.58 km² in size.
- The overall burned proportion of the buffer zone is calculated as: 37.64 km² ÷ 58.58 km² = 0.643 (64.3% burned).

Under the hypothesis that all vegetation types burn equally, we expect each vegetation type to have 64.3% of its area within the buffer zone burned.

To determine the burn area ratio for heathland, we compare the actual burned area with the expected burned area:

$$Burn area ratio = Actual burn area / Expected burn area$$

$$= 1.21 km^{2} / (0.643 \times1.29 km^{2})$$

$$= 1.21 / 0.829$$

$$= 1.46$$

Thus, the burn area ratio for heathland is 1.46×, indicating it burned disproportionately more than expected.

# Rationale for selected variables in the G-value metric

The G-value is a standardized metric integrating multiple genetic variables to quantify genetic diversity and structure within defined groups, such as sites or populations. Designed to align with Essential Biodiversity Variables (EBVs), it minimizes redundancy while capturing key dimensions of genetic diversity relevant to conservation. Variable selection was tailored to the dataset and study objectives, with alternative measures applicable in different contexts. A Spearman correlation analysis was conducted to assess relationships among variables and identify highly correlated ones, which were considered redundant and excluded from the final selection. The relationships between these metrics are presented in figure 3.1 below.

| **EBV** | **Variable** | **Included in G-value** | **Rationale** |
| --- | --- | --- | --- |
| Genetic diversity (evenness) | Heterozygosity (*H*_o_) | Yes | Heterozygosity is a fundamental aspect of genetic diversity and is explicitly referenced in CBD goals, making it essential for conservation prioritization. |
| Genetic diversity (richness) | Total common alleles | Yes | Allelic richness is another key component of genetic diversity highlighted in CBD goals. Including this ensures representation of the variety of alleles across sites. |
|  | Private common alleles | No | Private alleles were rare within our dataset. Including them would have added little value in this scenario but may be relevant in contexts with greater population differentiation. |
|  | Rare alleles | No | Rare alleles contribute to adaptive potential but were excluded here due to their rarity and high correlation with common alleles, making them redundant for the G-value in this case. |
| Genetic differentiation/ Inbreeding within site | Euclidean distance within site | Yes | This metric directly measures diversity within a site without reliance on a specific model (e.g., kinship or PCA). Greater Euclidean distance indicates higher diversity or less similarity among individuals. |
|  | Kinship within site | No | Kinship models often perform poorly in estimating relationships beyond close relatives, leading to inaccuracies. |
| Genetic differentiation between sites | Euclidean distance between sites | Yes | This provides a straightforward measure of differentiation among sites and avoids reliance on models. It is highly correlated with *F*_ST_, but more robust with smaller sample sizes. |
|  | *F*_ST_ | No | Although *F*_ST_ is widely used for measuring differentiation, it performs better with larger population sizes. Euclidean distance was preferred due to its high correlation with *F*_ST_ (0.516) and suitability for small samples. |
| Inbreeding | *F*_IS_ | No | *F*_IS_ was excluded because of its strong negative correlation with heterozygosity (-0.798 overall, <-0.95 within species). Its inclusion would have introduced redundancy without adding new information. |
| Population size | Effective population size (*N*_e_) | No | Estimating *N*_e_ was not feasible with the available data (SNPs not mapped to a reference genome) but could be very informative with other datasets. |
|  | Number of individuals (*n*) | Yes | The number of individuals is critical for maintaining viable, self-sustaining populations and is directly relevant to conservation efforts. |

**Figure 3.1:** Pairplot showing Spearman correlation coefficients among candidate genetic variables considered for inclusion in the final G-value calculation. Correlations are displayed for each species individually and across all species combined. Statistical significance is indicated by standard notation: . (p ≤ 0.1), * (p ≤ 0.05), ** (p ≤ 0.01), and *** (p ≤ 0.001). Variables include Kinship (estimated using PLINK IBD within genetic groups), mean Euclidean distances within and between sites, *F*_ST_ between sites (calculated using Weir and Hill), total alleles (TA), private alleles (PA), mean *H*_o_ (observed heterozygosity), mean *F*_IS_ (inbreeding coefficient), number of individuals (*n*), and the final G-value per management site.

# G-value calculation

The G-value is a composite metric designed to summarize multiple genetic variables into a single, standardized statistic for a defined group of individuals, such as those in a particular *in situ* site. By combining different measures of genetic diversity and structure, the G-value provides a practical and flexible tool for conservation and population studies.

## How the G-value is calculated

1. **Raw G-value calculation:** the raw G-value is calculated as the weighted sum of selected genetic variables. Each variable $v$ represents a distinct genetic metric (e.g., heterozygosity, allele counts), and $w$ is its user-defined weight, reflecting its importance in the analysis.

$$G\text{-}value \left( raw \right)={(w}_{1}\times v_{1})+ {(w}_{2}\times v_{2})+\ldots+{(w}_{n}\times v_{n})$$

2. **Final G-value normalization:** to ensure comparability across different species or datasets, the raw G-value is normalized by dividing it by the maximum raw G-value observed for any site within the species:

$$G\text{-}value \left( final \right)=\frac{G\text{-}value \left( raw \right)}{Max G\text{-}value in species \left( raw \right)}$$

## Variables we used in G-value calculation

To standardize comparisons, each variable is normalized relative to its maximum observed value. This ensures that all variables contribute proportionally to the final G-value, regardless of their original scales. The variables that we included are:

1. **Average heterozygosity:** represents the genetic variability within a site, normalized by dividing the site's average heterozygosity by the maximum average heterozygosity of any site.
2. **Proportion of common alleles:** measures the presence of widespread alleles in a site, normalized by dividing the number of common alleles in the site by the total number of common alleles in the species (within the defined dataset).
3. **Number of individuals (*n*):** accounts for the size of the site, normalized by dividing the number of individuals in the site by the largest site size.
4. **Mean genetic Euclidean distance within site:** quantifies genetic divergence among individuals within a site, normalized by dividing the site's mean Euclidean distance by the largest pairwise genetic distance between individuals in the dataset.
5. **Mean genetic Euclidean distance between sites:** measures genetic divergence between the focal site and others, normalized by dividing the mean inter-site Euclidean distance by the largest pairwise genetic distance in the dataset.

### Example of how Euclidean distances were calculated

This example demonstrates the calculation of mean normalized Euclidean distances within and between sites based on a filtered biallelic genotype matrix, where genotypes are encoded as 0 (AA), 1 (AB), and 2 (BB).

1. Euclidean distance calculation

Pairwise Euclidean distances are calculated for all individuals across sites. The resulting distance matrix is shown below, where rows and columns represent individuals grouped by site (A, B, or C).

| Site |  | A | A | A | B | B | C | C | C | C |
| --- | --- | --- | --- | --- | --- | --- | --- | --- | --- | --- |
|  | Individual | ID1 | ID2 | ID3 | ID4 | ID5 | ID6 | ID7 | ID8 | ID9 |
| A | ID1 | 0.0 | 41.3 | 45.3 | 41.2 | 40.0 | 42.3 | 59.0 | 56.5 | 53.9 |
| A | ID2 | 41.3 | 0.0 | 24.9 | 18.2 | 14.0 | 17.1 | 60.4 | 57.4 | 54.9 |
| A | ID3 | 45.3 | 24.9 | 0.0 | 26.4 | 23.6 | 26.4 | 63.2 | 62.0 | 59.8 |
| B | ID4 | 41.2 | 18.2 | 26.4 | 0.0 | 15.0 | 19.1 | 61.1 | 58.1 | 55.6 |
| B | ID5 | 40.0 | 14.0 | 23.6 | 15.0 | 0.0 | 15.3 | 59.4 | 56.8 | 54.3 |
| C | ID6 | 42.3 | 17.1 | 26.4 | 19.1 | 15.3 | 0.0 | 60.5 | 58.0 | 55.8 |
| C | ID7 | 59.0 | 60.4 | 63.2 | 61.1 | 59.4 | 60.5 | 0.0 | 59.4 | 57.2 |
| C | ID8 | 56.5 | 57.4 | 62.0 | 58.1 | 56.8 | 58.0 | 59.4 | 0.0 | 20.7 |
| C | ID9 | 53.9 | 54.9 | 59.8 | 55.6 | 54.3 | 55.8 | 57.2 | 20.7 | 0.0 |

1. Normalization

Each distance is normalized by dividing it by the largest pairwise distance in the dataset. This transforms the matrix into a normalized form, where distances are expressed as proportions of the maximum observed distance.

| Site |  | A | A | A | B | B | C | C | C | C |
| --- | --- | --- | --- | --- | --- | --- | --- | --- | --- | --- |
|  | Individual | ID1 | ID2 | ID3 | ID4 | ID5 | ID6 | ID7 | ID8 | ID9 |
| A | ID1 | 0.000 | 0.654 | 0.716 | 0.652 | 0.634 | 0.670 | 0.933 | 0.895 | 0.854 |
| A | ID2 | 0.654 | 0.000 | 0.395 | 0.289 | 0.222 | 0.271 | 0.956 | 0.909 | 0.870 |
| A | ID3 | 0.716 | 0.395 | 0.000 | 0.418 | 0.374 | 0.418 | 1.000 | 0.981 | 0.947 |
| B | ID4 | 0.652 | 0.289 | 0.418 | 0.000 | 0.237 | 0.302 | 0.966 | 0.920 | 0.880 |
| B | ID5 | 0.634 | 0.222 | 0.374 | 0.237 | 0.000 | 0.242 | 0.941 | 0.899 | 0.859 |
| C | ID6 | 0.670 | 0.271 | 0.418 | 0.302 | 0.242 | 0.000 | 0.958 | 0.918 | 0.883 |
| C | ID7 | 0.933 | 0.956 | 1.000 | 0.966 | 0.941 | 0.958 | 0.000 | 0.941 | 0.905 |
| C | ID8 | 0.895 | 0.909 | 0.981 | 0.920 | 0.899 | 0.918 | 0.941 | 0.000 | 0.328 |
| C | ID9 | 0.854 | 0.870 | 0.947 | 0.880 | 0.859 | 0.883 | 0.905 | 0.328 | 0.000 |

1. Calculation of Mean Distances

The mean Euclidean distance is computed separately for individuals within a site and between sites.

- **Within Site A:** The mean of all pairwise distances between individuals within site A is = (0.654 + 0.716 + 0.395) / 3 = 0.588
- **Between Sites (A vs others):** The mean of all pairwise distances between individuals in site A and individuals in other sites is = (0.652 + 0.289 + … +0.328) / 33 =0.711

### Example of how G-value was calculated

This example demonstrates how we calculated G-values, using fictional data.

1. Gather raw values for each *in situ* site

The raw metrics include mean Euclidean distances within and between sites, mean heterozygosity (*H*_o_), total common alleles (TA), and the number of individuals (*n*) per site.

| Site | dist within | dist between | mean *H*_o_ | TA | n |
| --- | --- | --- | --- | --- | --- |
| A | 0.588 | 0.711 | 0.19 | 1182 | 3 |
| B | 0.237 | 0.714 | 0.17 | 1006 | 2 |
| C | 0.822 | 0.677 | 0.20 | 1214 | 4 |

1. Normalize values

To standardize the metrics across sites, normalization is applied to ensure all values fall within the range of 0 to 1. Each variable is normalized differently, as outlined below:

- **Euclidean distances**: Already normalized during initial calculations.
- **Mean Ho**: Divided by the largest site mean *H*_o_ (in this case Site C is max *H*_o_ = 0.2).
- **Total alleles (TA)**: Divided by the total number of alleles observed across the species (in this case we use 1400).
- **Number of individuals (*n*)**: Divided by the largest site size (in this case Site C is max *n* = 4).

| Site | dist within | dist between | normalized *H*_o_ | normalized TA | normalized *n* |
| --- | --- | --- | --- | --- | --- |
| A | 0.588 | 0.711 | 0.95 | 0.84 | 0.75 |
| B | 0.237 | 0.714 | 0.85 | 0.72 | 0.5 |
| C | 0.822 | 0.677 | 1.00 | 0.87 | 1 |

1. Calculate raw G-value
2. The raw G-value is calculated as the weighted sum of selected genetic variables. Here, all variables were weighted equally.

$$G\text{-}value \left( raw \right)={(w}_{1}\times v_{1})+ {(w}_{2}\times v_{2})+\ldots+{(w}_{n}\times v_{n})$$

3. The final G-value is normalised by dividing by the largest raw G-value of the species.

$$G\text{-}value \left( final \right)=\frac{G\text{-}value \left( raw \right)}{Max G\text{-}value in species \left( raw \right)}$$

| Site | raw G-value | final G-value |
| --- | --- | --- |
| A | 0.59+0.71+0.95+0.84+0.75 = **3.844** | 3.844/4.366 = **0.88** |
| B | 0.24+0.71+0.85+0.72+0.5 = **3.020** | 3.02/4.366 = **0.69** |
| C | 0.82+0.68+1+0.87+1 = **4.366** | 4.366/4.366 = **1.00** |

The final G-values represent a relative measure of genetic value incorporating genetic diversity, evenness, and differentiation.

# Generalized linear model results

Generalized Linear Models (GLMs) were used to analyse genetic diversity metrics across vegetation types, as they are well-suited for the characteristics of our dataset. GLMs accommodate non-normal data distributions, uneven sample sizes, and the inclusion of multiple predictors, such as species, vegetation type, and sample size, to estimate effect sizes and assess statistical significance. Below, we present the detailed results of these analyses.

The Estimate values in the GLM output represent the magnitude and direction of the relationship between each predictor variable (e.g., species or vegetation type) and the response variable (heterozygosity or allele count), with positive values indicating an increase and negative values indicating a decrease in the response. The endangered target species are indicated (☆).

## Total common alleles

glm(formula = TA ~ Species: + Vegetation + n, data = common_alleles)

(Dispersion parameter for gaussian family taken to be 0.006800024)

Null deviance: 1.0111 on 70 degrees of freedom

Residual deviance: 0.3468 on 51 degrees of freedom

AIC: -134.35

Number of Fisher Scoring iterations: 2

| Predictor | Estimate | Std. Error | t value | Pr(>|t|) |
| --- | --- | --- | --- | --- |
| (Intercept) | 0.719 | 0.099 | 7.29 | 1.88E-09 |
| Species: Ceratopetalum apetalum | 0.024 | 0.051 | 0.47 | 0.640 |
| Species: Diploglottis australis | -0.018 | 0.052 | -0.34 | 0.735 |
| Species: Doryphora sassafras | -0.057 | 0.051 | -1.10 | 0.275 |
| Species: Eidothea hardeniana ☆ | -0.049 | 0.053 | -0.93 | 0.358 |
| Species: Elaeocarpus sedentarius ☆ | 0.023 | 0.051 | 0.44 | 0.660 |
| Species: Hicksbeachia pinnatifolia | -0.044 | 0.053 | -0.83 | 0.408 |
| Species: Neolitsea dealbata | -0.018 | 0.052 | -0.35 | 0.730 |
| Species: Schizomeria ovata | 0.031 | 0.054 | 0.57 | 0.570 |
| Species: Sloanea australis | -0.029 | 0.052 | -0.56 | 0.578 |
| Species: Sloanea woollsii | -0.064 | 0.053 | -1.20 | 0.234 |
| Species: Uromyrtus australis ☆ | -0.050 | 0.050 | -0.99 | 0.328 |
| Vegetation: Heathlands | -0.060 | 0.128 | -0.47 | 0.638 |
| Vegetation: Rainforests (combined) | 0.197 | 0.097 | 2.03 | 0.048 |
| Vegetation: Rainforests (Pyrophyte 0-<1%) | 0.192 | 0.095 | 2.03 | 0.047 |
| Vegetation: Rainforests (Pyrophyte 1%-10%) | 0.221 | 0.094 | 2.36 | 0.022 |
| Vegetation: Rainforests (Pyrophyte 11%-30%) | 0.070 | 0.093 | 0.75 | 0.455 |
| Vegetation: Wet Sclerophyll Forests (Grassy sub-formation) | 0.095 | 0.093 | 1.03 | 0.310 |
| Vegetation: Wet Sclerophyll Forests (Shrubby sub-formation) | 0.180 | 0.093 | 1.93 | 0.059 |
| number of individuals (n) | 0.002 | 0.001 | 3.00 | 0.004 |

## Total rare alleles

glm(formula = TA ~ Species: + Vegetation + n, data = rare_alleles)

(Dispersion parameter for gaussian family taken to be 0.006144608)

Null deviance: 1.83497 on 70 degrees of freedom

Residual deviance: 0.31338 on 51 degrees of freedom

AIC: -141.55

Number of Fisher Scoring iterations: 2

| Predictor | Estimate | Std. Error | t value | Pr(>|t|) |
| --- | --- | --- | --- | --- |
| (Intercept) | 0.515 | 0.094 | 5.49 | 1.27E-06 |
| Species: Ceratopetalum apetalum | 0.003 | 0.048 | 0.06 | 0.953 |
| Species: Diploglottis australis | -0.024 | 0.050 | -0.49 | 0.627 |
| Species: Doryphora sassafras | -0.037 | 0.049 | -0.75 | 0.458 |
| Species: Eidothea hardeniana ☆ | -0.002 | 0.050 | -0.04 | 0.965 |
| Species: Elaeocarpus sedentarius ☆ | -0.039 | 0.049 | -0.81 | 0.422 |
| Species: Hicksbeachia pinnatifolia | -0.058 | 0.050 | -1.16 | 0.252 |
| Species: Neolitsea dealbata | -0.035 | 0.049 | -0.71 | 0.483 |
| Species: Schizomeria ovata | 0.032 | 0.051 | 0.64 | 0.528 |
| Species: Sloanea australis | -0.052 | 0.049 | -1.05 | 0.299 |
| Species: Sloanea woollsii | -0.024 | 0.051 | -0.47 | 0.643 |
| Species: Uromyrtus australis ☆ | -0.021 | 0.048 | -0.44 | 0.659 |
| Vegetation: Heathlands | 0.005 | 0.122 | 0.04 | 0.970 |
| Vegetation: Rainforests (combined) | 0.273 | 0.092 | 2.95 | 0.005 |
| Vegetation: Rainforests (Pyrophyte 0-<1%) | 0.164 | 0.090 | 1.83 | 0.074 |
| Vegetation: Rainforests (Pyrophyte 1%-10%) | 0.239 | 0.089 | 2.68 | 0.010 |
| Vegetation: Rainforests (Pyrophyte 11%-30%) | 0.058 | 0.088 | 0.65 | 0.516 |
| Vegetation: Wet Sclerophyll Forests (Grassy sub-formation) | 0.082 | 0.088 | 0.92 | 0.361 |
| Vegetation: Wet Sclerophyll Forests (Shrubby sub-formation) | 0.129 | 0.088 | 1.46 | 0.150 |
| number of individuals (n) | 0.003 | 0.001 | 5.15 | 4.26E-06 |

## Private common alleles

glm(formula = PA ~ Species: + Vegetation + n, data = common_alleles)

(Dispersion parameter for gaussian family taken to be 0.0004268585)

Null deviance: 0.053135 on 70 degrees of freedom

Residual deviance: 0.021770 on 51 degrees of freedom

AIC: -330.89

Number of Fisher Scoring iterations: 2

| Predictor | Estimate | Std. Error | t value | Pr(>|t|) |
| --- | --- | --- | --- | --- |
| (Intercept) | 1.3E-02 | 2.5E-02 | 0.52 | 0.61 |
| Species: Ceratopetalum apetalum | -1.1E-02 | 1.3E-02 | -0.87 | 0.39 |
| Species: Diploglottis australis | 9.9E-03 | 1.3E-02 | 0.75 | 0.45 |
| Species: Doryphora sassafras | 8.1E-03 | 1.3E-02 | 0.63 | 0.53 |
| Species: Eidothea hardeniana ☆ | -1.0E-02 | 1.3E-02 | -0.76 | 0.45 |
| Species: Elaeocarpus sedentarius ☆ | -1.1E-02 | 1.3E-02 | -0.86 | 0.39 |
| Species: Hicksbeachia pinnatifolia | -7.5E-03 | 1.3E-02 | -0.56 | 0.58 |
| Species: Neolitsea dealbata | -7.9E-04 | 1.3E-02 | -0.06 | 0.95 |
| Species: Schizomeria ovata | -1.3E-02 | 1.3E-02 | -0.95 | 0.35 |
| Species: Sloanea australis | 8.3E-03 | 1.3E-02 | 0.64 | 0.52 |
| Species: Sloanea woollsii | 1.8E-02 | 1.3E-02 | 1.32 | 0.19 |
| Species: Uromyrtus australis ☆ | -8.8E-03 | 1.3E-02 | -0.70 | 0.49 |
| Vegetation: Heathlands | -3.0E-02 | 3.2E-02 | -0.95 | 0.35 |
| Vegetation: Rainforests (combined) | 4.1E-02 | 2.4E-02 | 1.70 | 0.10 |
| Vegetation: Rainforests (Pyrophyte 0-<1%) | -1.0E-02 | 2.4E-02 | -0.44 | 0.66 |
| Vegetation: Rainforests (Pyrophyte 1%-10%) | -2.5E-03 | 2.3E-02 | -0.11 | 0.92 |
| Vegetation: Rainforests (Pyrophyte 11%-30%) | -9.2E-03 | 2.3E-02 | -0.40 | 0.69 |
| Vegetation: Wet Sclerophyll Forests (Grassy sub-formation) | -1.0E-02 | 2.3E-02 | -0.44 | 0.66 |
| Vegetation: Wet Sclerophyll Forests (Shrubby sub-formation) | -1.0E-02 | 2.3E-02 | -0.43 | 0.67 |
| number of individuals (n) | -1.9E-05 | 1.6E-04 | -0.12 | 0.90 |

## Private rare alleles

glm(formula = PA ~ Species: + Vegetation + n, data = rare_alleles)

(Dispersion parameter for gaussian family taken to be 0.004092144)

Null deviance: 0.86544 on 70 degrees of freedom

Residual deviance: 0.20870 on 51 degrees of freedom

AIC: -170.41

Number of Fisher Scoring iterations: 2

| Predictor | Estimate | Std. Error | t value | Pr(>|t|) |
| --- | --- | --- | --- | --- |
| (Intercept) | 0.025 | 0.076 | 0.33 | 0.75 |
| Species: Ceratopetalum apetalum | -0.021 | 0.039 | -0.54 | 0.59 |
| Species: Diploglottis australis | 0.064 | 0.041 | 1.59 | 0.12 |
| Species: Doryphora sassafras | 0.056 | 0.040 | 1.39 | 0.17 |
| Species: Eidothea hardeniana ☆ | -0.087 | 0.041 | -2.11 | 0.04 |
| Species: Elaeocarpus sedentarius ☆ | 0.005 | 0.040 | 0.12 | 0.90 |
| Species: Hicksbeachia pinnatifolia | 0.039 | 0.041 | 0.95 | 0.35 |
| Species: Neolitsea dealbata | 0.061 | 0.040 | 1.52 | 0.14 |
| Species: Schizomeria ovata | -0.022 | 0.042 | -0.52 | 0.61 |
| Species: Sloanea australis | 0.067 | 0.040 | 1.66 | 0.10 |
| Species: Sloanea woollsii | 0.055 | 0.041 | 1.33 | 0.19 |
| Species: Uromyrtus australis ☆ | -0.037 | 0.039 | -0.95 | 0.35 |
| Vegetation: Heathlands | -0.078 | 0.099 | -0.79 | 0.44 |
| Vegetation: Rainforests (combined) | 0.181 | 0.075 | 2.40 | 0.02 |
| Vegetation: Rainforests (Pyrophyte 0-<1%) | 0.006 | 0.073 | 0.08 | 0.94 |
| Vegetation: Rainforests (Pyrophyte 1%-10%) | 0.068 | 0.073 | 0.93 | 0.36 |
| Vegetation: Rainforests (Pyrophyte 11%-30%) | -0.030 | 0.072 | -0.42 | 0.68 |
| Vegetation: Wet Sclerophyll Forests (Grassy sub-formation) | -0.019 | 0.072 | -0.27 | 0.79 |
| Vegetation: Wet Sclerophyll Forests (Shrubby sub-formation) | -0.018 | 0.072 | -0.25 | 0.81 |
| number of individuals (n) | 0.001 | 0.000 | 1.59 | 0.12 |

## Heterozygosity

glm(formula = Heterozygosity ~ Vegetation + Species, data = data)

Null deviance: 2.0923 on 922 degrees of freedom

Residual deviance: 1.0228 on 905 degrees of freedom

AIC: -3623.7

Number of Fisher Scoring iterations: 2

| **Predictor** | **Estimate** | **Std. Error** | **t value** | **Pr(>|t|)** |  |
| --- | --- | --- | --- | --- | --- |
| (Intercept) | 0.168 | 0.020 | 8.32 | 3.32e-16 | *** |
| Vegetation: Heathlands | 0.036 | 0.039 | 0.91 | 0.37 |  |
| Vegetation: Rainforests (Pyrophyte 0 <1%) | 0.031 | 0.020 | 1.54 | 0.12 |  |
| Vegetation: Rainforests (Pyrophyte 1% 10%) | 0.025 | 0.020 | 1.28 | 0.20 |  |
| Vegetation: Rainforests (Pyrophyte 11% 30%) | 0.003 | 0.020 | 0.17 | 0.87 |  |
| Vegetation: Wet Sclerophyll Forests (Grassy sub formation) | 0.030 | 0.020 | 1.48 | 0.14 |  |
| Vegetation: Wet Sclerophyll Forests (Shrubby sub formation) | 0.018 | 0.020 | 0.89 | 0.37 |  |
| Species: Ceratopetalum apetalum | 0.033 | 0.005 | 6.41 | 2.40e-10 | *** |
| Species: Diploglottis australis | 0.070 | 0.007 | 10.28 | < 2e-16 | *** |
| Species: Doryphora sassafras | 0.022 | 0.006 | 3.82 | 0.00 | *** |
| Species: Eidothea hardeniana ☆ | 0.048 | 0.004 | 10.98 | < 2e-16 | *** |
| Species: Elaeocarpus sedentarius ☆ | 0.025 | 0.005 | 4.67 | 3.48e-06 | *** |
| Species: Hicksbeachia pinnatifolia | -0.009 | 0.007 | -1.21 | 0.23 |  |
| Species: Neolitsea dealbata | 0.028 | 0.006 | 4.50 | 7.61e-06 | *** |
| Species: Schizomeria ovata | -0.001 | 0.006 | -0.25 | 0.80 |  |
| Species: Sloanea australis | 0.080 | 0.006 | 12.91 | < 2e-16 | *** |
| Species: Sloanea woollsii | 0.040 | 0.006 | 7.01 | 4.77e-12 | *** |
| Species: Uromyrtus australis ☆ | -0.048 | 0.005 | -10.19 | < 2e-16 | *** |

Estimated marginal means (EMMEANS) of heterozygosity for each species based on the GLM. Values include the estimated mean heterozygosity (emmean), standard error (SE), degrees of freedom (df), and 95% confidence intervals (lower.CL, upper.CL)

| **Species** | **emmean** | **SE** | **df** | **lower.CL** | **upper.CL** |
| --- | --- | --- | --- | --- | --- |
| Argyrodendron trifoliolatum | 0.188 | 0.007 | 905 | 0.175 | 0.201 |
| Ceratopetalum apetalum | 0.221 | 0.007 | 905 | 0.208 | 0.234 |
| Diploglottis australis | 0.258 | 0.008 | 905 | 0.242 | 0.274 |
| Doryphora sassafras | 0.211 | 0.007 | 905 | 0.196 | 0.225 |
| Eidothea hardeniana ☆ | 0.236 | 0.006 | 905 | 0.224 | 0.249 |
| Elaeocarpus sedentarius ☆ | 0.214 | 0.007 | 905 | 0.2 | 0.227 |
| Hicksbeachia pinnatifolia | 0.18 | 0.008 | 905 | 0.163 | 0.196 |
| Neolitsea dealbata | 0.216 | 0.008 | 905 | 0.201 | 0.231 |
| Schizomeria ovata | 0.187 | 0.007 | 905 | 0.174 | 0.2 |
| Sloanea australis | 0.268 | 0.008 | 905 | 0.253 | 0.283 |
| Sloanea woollsii | 0.228 | 0.007 | 905 | 0.215 | 0.242 |
| Uromyrtus australis ☆ | 0.141 | 0.006 | 905 | 0.128 | 0.153 |

# Koala example

To illustrate the application of the clustering method on different data with area-based constraints, we used publicly available data from the Atlas of Living Australia on koala (*Phascolarctos cinereus* [Goldfuss, 1817]) sightings in New South Wales from 2022 to 2025.

Given the larger scale of this dataset (with many more observations), we adjusted the initial DBSCAN clustering parameters to require a minimum of 100 observations to form a cluster and set an epsilon value of 5 km, defining the maximum distance for including a point in a cluster. For subsequent k-means subdivision of the DBSCAN clusters, we applied a maximum area constraint of 200 km².

This example highlights the method’s applicability over a broad geographic area to identify regions of high observation density and demonstrates the use of area-based constraints rather than perimeter-based ones. It also underscores how public observation data can introduce bias, with high-density observations often clustered around populated areas—such as the southern Sydney and the Northern Rivers region of NSW.

While we argue that this method is particularly suited for species with survey data due to its ability to reduce bias, this example is valuable for demonstrating how scale and parameter adjustments influence the outcomes.


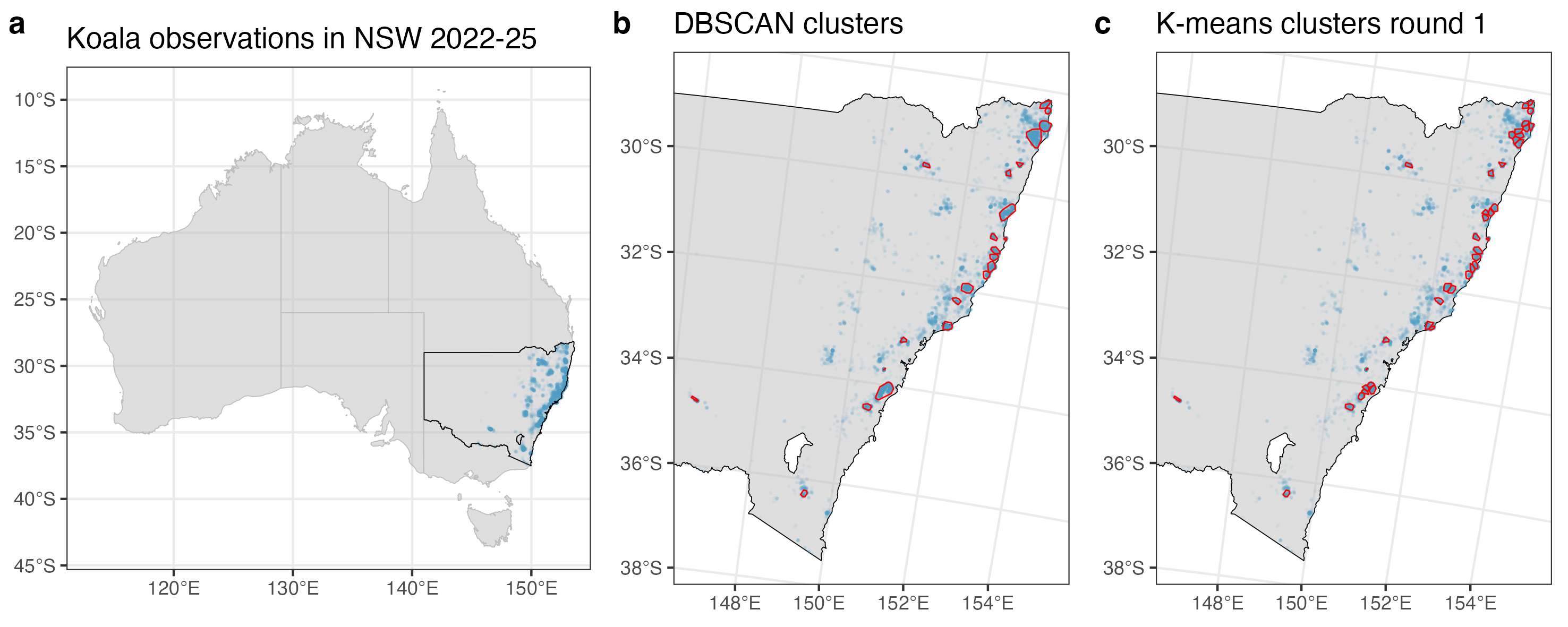


**Figure 6.1:** Koala clustering example. (A) Atlas of Living Australia (ALA) koala observations (blue points) across New South Wales from 2022 to 2025. (B) DBSCAN clusters of koala observations. The orange arrow highlights a cluster centred around southern Sydney, while the yellow arrow points to the Northern Rivers region. (C) k-means clusters derived from the subdivision of DBSCAN clusters, constrained to a maximum area of 200 km².
